# Early spatiotemporal-specific changes in intermediate signals are predictive of cytotoxic sensitivity to TNFα and co-treatments

**DOI:** 10.1038/srep43541

**Published:** 2017-03-08

**Authors:** Lit-Hsin Loo, Nicola Michelle Bougen-Zhukov, Wei-Ling Cecilia Tan

**Affiliations:** 1 Bioinformatics Institute, Agency for Science, Technology and Research, 30 Biopolis Street, #07-01 Matrix, Singapore 138671, Singapore

## Abstract

Signaling pathways can generate different cellular responses to the same cytotoxic agents. Current quantitative models for predicting these differential responses are usually based on large numbers of intracellular gene products or signals at different levels of signaling cascades. Here, we report a study to predict cellular sensitivity to tumor necrosis factor alpha (TNFα) using high-throughput cellular imaging and machine-learning methods. We measured and compared 1170 protein phosphorylation events in a panel of human lung cancer cell lines based on different signals, subcellular regions, and time points within one hour of TNFα treatment. We found that two spatiotemporal-specific changes in an intermediate signaling protein, p90 ribosomal S6 kinase (RSK), are sufficient to predict the TNFα sensitivity of these cell lines. Our models could also predict the combined effects of TNFα and other kinase inhibitors, many of which are not known to target RSK directly. Therefore, early spatiotemporal-specific changes in intermediate signals are sufficient to represent the complex cellular responses to these perturbations. Our study provides a general framework for the development of rapid, signaling-based cytotoxicity screens that may be used to predict cellular sensitivity to a cytotoxic agent, or identify co-treatments that may sensitize or desensitize cells to the agent.

Many cytotoxic agents, including cytokines, drugs, and toxicants, rapidly induce the phosphorylation of a common set of intermediate signaling proteins that drive diverse types of downstream effectors^1^,^2^,^3^. The rapid activations of these signaling proteins (often within minutes) make them attractive markers for testing primary cells that cannot tolerate prolonged *in vitro* culture. However, these signaling proteins may be involved in the generation of different phenotypic outcomes^4^,^5^, thus making accurate prediction of these outcomes very challenging. To predict the sensitivity of human cells to a cytotoxic agent, most current quantitative models are based on the abundance or modification levels of large numbers of gene products measured from the entire cellular regions or extracts and/or at different levels of signaling cascades. For example, hundreds to thousands of protein phosphorylation events measured from tens of signaling proteins, which include receptors, kinases, transcription factors, and caspases, from whole-cell extracts or regions have been used to predict apoptotic responses of human cancer cell lines^1^,^6^. Genome-wide measurements of basal genetic status or gene expression levels have also been used^7^,^8^. However, the contributions of the individual components of these high-dimensional models cannot be easily determined. It is often unclear at which level of the signaling cascades that signal divergence first occurs, and whether individual signals are sufficient to predict the eventual phenotypic outcomes. Furthermore, the ability of these previous models to predict the effects of new co-treatments, such as small-molecule kinase inhibitors, that can sensitive or de-sensitize cells to cytotoxic agents is often untested. Therefore, the complex relationships between intracellular signals and differential cellular responses to the same cytotoxic agents remain poorly understood. Recently, a quantitative model based on the temporal dynamics of caspases 8 activation was developed to predict fractional killing of cancer cells treated with a cytotoxic agent, tumor necrosis factor-related apoptosis inducing ligand (TRAIL)^9^. This suggests the possibility of building highly predictive models based on very small numbers of readouts by exploiting the temporal or even spatial information in cellular responses to cytotoxic agents.

Here, we report a study of the signal transduction cascades and cell-death responses induced by tumor necrosis factor alpha (TNFα) in eight human non-small cell lung cancer (NSCLC) cell lines with different levels of TNFα sensitivity. TNFα is a death receptor ligand and induces signaling cascades that mediate inflammatory, proliferative, and/or cell-death responses^10^. Our goal was to build signaling-based computational models that can predict cytotoxic sensitivity to TNFα. We hypothesize that signals at or near the divergent points of TNFα signaling cascades can be used as surrogate markers of TNFα-induced cytotoxicity. Consequently, computational models based on these signals may predict the eventual effects of TNFα and co-treatments, even though these co-treatments may not directly affect the signals. Although signals that give the most predictive models are likely to be involved in TNFα response, they are not necessary regulators of TNFα sensitivity. For example, the phosphorylation levels of the substrates of a hypothetical regulator may better reflect the regulator’s activity than the phosphorylation levels of the regulator itself. Identifying regulators of TNFα sensitivity was not a main goal of our study.

Our study has three major differences compared to other previous work with similar goals. The first difference is that we systematically measured thirteen intracellular signals, and compared the ability of each individual signal in predicting cellular sensitivity to TNFα. These signals include site-specific phosphorylations of eight intermediate protein kinases and five downstream effectors, which form a network of signaling cascades (Fig. 1A). We selected these signals because most of them are known to be induced by TNFα and/or other cytotoxic agents (Supplementary Table S1). The second difference is that we quantified changes in these signals at nine different subcellular regions and ten different time points within one hour of TNFα treatment using single-cell imaging and automated image analysis methods developed by us^11^,^12^,^13^,^14^. These high-resolution spatiotemporal measurements of signaling events helped us to identify signaling differences between sensitive and resistant cells that are not obvious from measurements based on whole-cell regions/extracts or coarser time points. A linear regression model based on the signaling events was then constructed using an elastic net algorithm^15^ to predict cellular sensitivity to TNFα. Lastly, we experimentally tested the regression model using four small-molecule kinase inhibitors that target different components of the TNFα signaling network. These inhibitors were TPCA-1 (for IκB kinase beta, IKKβ), SP600125 (for c-Jun N-terminal kinases, JNK), BI-D1870 (for RSK), and SB202190 (for p38 mitogen activated protein kinase, p38). This is a very stringent test of our model because the model was constructed without using any information from these inhibitors. These three differences allow us to show that early spatiotemporal changes in intermediate signals are predictive of cytotoxic sensitivity to TNFα and co-treatments.

## Results

### Lung cancer cells have differential TNFα sensitivity

**Figure 1:**
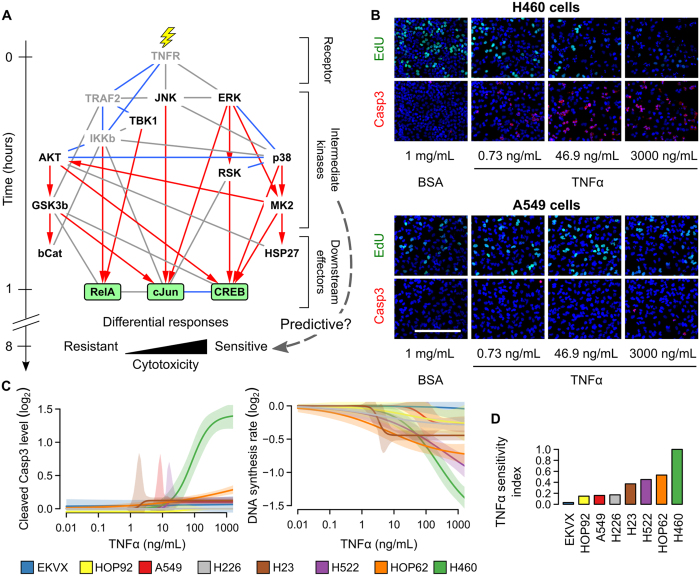
Human NSCLC cells have differential TNFα sensitivity. (**A**) Schematic showing the TNFα signaling network and the goal of our study. Changes in the phosphorylation and subcellular localization of thirteen intracellular signals (bold items) within one hour of TNFα treatment were studied for their abilities to predict cellular responses to TNFα. The network connections are based on the STRING database^50^ (gray lines = known functional associations, blue lines = known physical interactions, red arrows = known phosphorylation interactions, green boxes = transcription factors). (**B**) Immunofluorescence images showing the 5-ethynyl-2′-deoxyuridine (EdU) and cleaved caspase-3 (Casp3) staining of H460 and A549 cells treated with 0–3000 ng/mL of TNFα for eight hours (blue = DNA, green = EdU, red = Casp3, scale bar = 400 μm). Bovine serum albumin (BSA) was used as a negative control. Images from the same rows have the same exposure times and display intensity ranges. (**C**) The cleaved caspase-3 levels and DNA synthesis rates of eight NSCLC cell lines were measured after 8 hours of TNFα treatment (0–3000 ng/mL) in three replicates. The dose response curves of these readouts were fitted using standard log-logistic models. The shaded areas are 95th percentile confidence bands of the fitted response curves as determined using the drc library (Methods). (**D**) For each cell line, a final TNFα sensitivity index was determined by averaging the areas under the dose response curves of these two readouts. All the index values were normalized by dividing them with the maximum index value across all cell lines.

We first determined to what extent NSCLC cells respond differentially to TNFα. We obtained eight human NSCLC cell lines from the well-characterized NCI-60 cell line panel^16^. After treating the cell lines with TNFα for eight hours, we measured changes in two cytotoxic readouts: the synthesis rate of DNA and cleavage of caspase 3, a critical proteolytic enzyme involved in apoptosis^17^ (Fig. 1B and C). We found that DNA synthesis rate and cleaved-caspase 3 level are highly (and negatively) correlated to each other, but DNA synthesis rate is better in distinguishing cell lines with low cleaved-caspase 3 levels (Fig. 1C). Therefore, DNA synthesis rate provides important complementary information to cleaved-caspase 3 level. For each cell line, we computed a TNFα sensitivity index by averaging the areas under the dose response curves based on these two readouts (Supplementary Table S2). We found that these cell lines responded differentially to TNFα (Fig. 1D). The difference between the TNFα sensitivity indices of the most sensitive and resistant cell lines was >30 fold. Therefore, these cell lines provided an appropriate biological model system for us to compare the performances of different signaling or genomic features in predicting TNFα sensitivity.

The mutation status of several common cancer genes in these cell lines was previously determined^18^. We obtained data for three of the most frequently mutated genes in lung cancers, namely tumor protein p53 (TP53, mutated in ~40–50% of NSCLC^19^,^20^), epidermal growth factor receptor (EGFR, mutated in ~10–50% of NSCLC^21^), and phosphatase and tensin homolog (PTEN, mutated in ~5% of NSCLC^19^) (Supplementary Table S3). We found that p53 is mutated in five of the cell lines (except for A549 and H460), and EGFR and PTEN are wild types in all the cell lines (Supplementary Table S3). There is no clear association of mutations in these genes with TNFα sensitivity in the cell lines.

To efficiently search for the most predictive signals across all cell lines, we used a two-stage heuristic. First, the thirteen candidate signals were screened in two prototypic sensitive and resistant cell lines, namely H460 and A549. These cell lines were chosen because the sensitivity of H460 was ~six fold higher than of A549, and both of them have wildtype p53 (Supplementary Table S3) and similar doubling times. Then, a reduced set of candidate signals were selected and tested in all cell lines.

### Automated quantification of signaling responses

**Figure 2:**
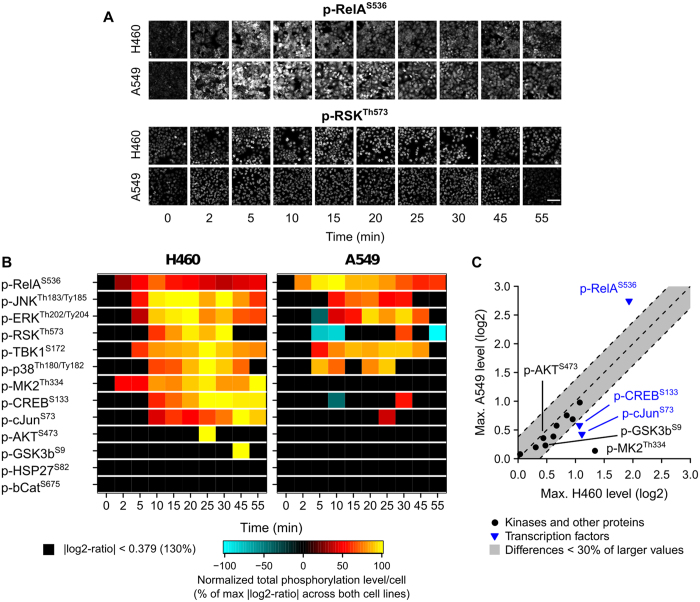
Intermediate kinase signals are commonly induced in sensitive and resistant cells. (**A**) Immunofluorescence images showing p-RelA^S536^ or p-RSK^Th573^ staining in H460 and A549 cells treated with 300 ng/mL of TNFα for 0 to 55 min (scale bar = 200 μm). Images for the same signals have the same exposure times and display intensity ranges. (**B**) Heatmaps showing changes in the total phosphorylation levels of the thirteen signals in H460 and A549 cells treated with 300 ng/mL of TNFα for 0 to 55 min. The values for all phosphorylation events are log_2_ ratios of their corresponding values at time 0 (without TNFα treatment). For visualization in this figure only, the log_2_ ratios for each signal were divided by their maximum absolute value across both cell lines. All changes less than 30% were colored in black. (**C**) Scatter plot showing the maximum levels of the thirteen signals in A549 and H460 cells. Signals in the gray region have <30% differences between their maximum values in the two cell lines.

In the first stage of our heuristic search, we treated H460 and A549 cells with 300 ng/mL TNFα or 0.1% bovine serum albumin (BSA, a negative control for the addition of proteins) for 10 different time points (between 0 to 55 min). Each time point was assayed in a different well on a 384-well microtiter plate. Then, we fixed the cells and used immunofluorescence microscopy to label and image the 13 candidate signals (Fig. 2A and Supplementary Fig. S1). After segmenting individual cells using automated image processing algorithms (Supplementary Fig. S2), we quantified the total intra-cellular levels of these signals for every individual cell, and computed the mean values of the signals across ~1000–4000 cells for each time point (Fig. 2B).

To minimize the effects of systemic errors, such as experimental variation and image processing artifacts, the following data analysis and quality control procedures were used. First, we detected cellular and nuclear regions solely based on mitochondria and DNA stains, respectively, independent of the phospho-protein stains (Supplementary Fig. S2). We found that the areas of the detected cellular regions have low variations across different time points (median coefficient of variation = ~5%, Supplementary Fig. S3). Second, we removed wells with low numbers of cells (usually <100 cells/well) from further analysis. Third, we observed that the signaling responses measured from the BSA-treated wells were relatively low and constant across different time points for all the measured signals (median coefficient of variation = ~10%, Supplementary Fig. S4). We found no evidence that our experimental or analysis procedures induced non-specific changes in most of the signals. Therefore, we simply normalized all the quantified signal values by taking their log_2_ ratios with respect to their values at time 0. In addition to standard statistical tests for reproducibility, we also required all differences in signaling responses to be larger than 30% (or 0.379 in the log_2_ scale), which was about three times the median coefficient-of-variation value for the BSA controls (Supplementary Fig. S4). Fourth, we performed the assays in duplicates and found that most of the duplicates were highly reproducible (Supplementary Figs S3 and S4). Therefore, we used their mean values for further analysis. Taken together, we conclude that the signaling response data generated from our imaging assays were highly reliable and reproducible.

### Most kinase signals are commonly induced

Can the observed differential TNFα responses be explained by the activation or inhibition of individual signals? We found that protein phosphorylation occurred rapidly within an hour, with most kinase signals peaking before transcription-factor signals (Fig. 2B). The only exception was the phosphorylation level of nuclear factor (NF)-κB p65 subunit at serine 536 (p-RelA^S536^), which peaked ~10 min after treatment. Among all the signals, transcription factors consistently had the highest absolute differences in their maximum phosphorylation levels across the two prototypic cell lines (Fig. 2C). Specifically, p-RelA^S536^ was higher (~175%) in resistant A549 cells, whereas the phosphorylation levels of Jun subunit of activating protein-1 transcription factor at serine 73 (p-cJun^S73^) and cAMP response element-binding protein at serine 133 (p-CREB^S133^) were higher (~161% and 140%, respectively) in sensitive H460 cells (Fig. 2C). These results agree with the known roles of RelA and JNK in attenuating^22^ or potentiating^23^ TNFα-induced cell death, respectively. These results also suggest that different transcriptional programs had been initiated in these two cell lines. In contrast, most of the tested kinase signals achieved similar maximum levels across both cell lines (with <30% differences, Fig. 2C). Some of these kinase signals, which include the phosphorylation levels of protein kinase B at serine 473 (p-AKT^S473^) and glycogen synthase kinase 3 beta at serine 9 (p-GSK3b^S9^), had relatively low values in both cell lines (Fig. 2C). Control experiments using another cytokine, insulin-like growth factor 1 (IGF1), showed that our assays could detect changes in these signals if they were induced (Supplementary Fig. S5). Taken together, the observed differential responses cannot simply be explained by the turning on or off of the individual kinases. Most kinase signals were either commonly induced or irresponsive to TNFα in both the sensitive and resistant cell lines. The understanding of this phenomenon, which is a hallmark of complex biomolecular systems^4^, may require further characterization of the spatiotemporal dynamics of the signaling responses.

### Most signals peak at different subcellular regions in sensitive and resistant cells

**Figure 3:**
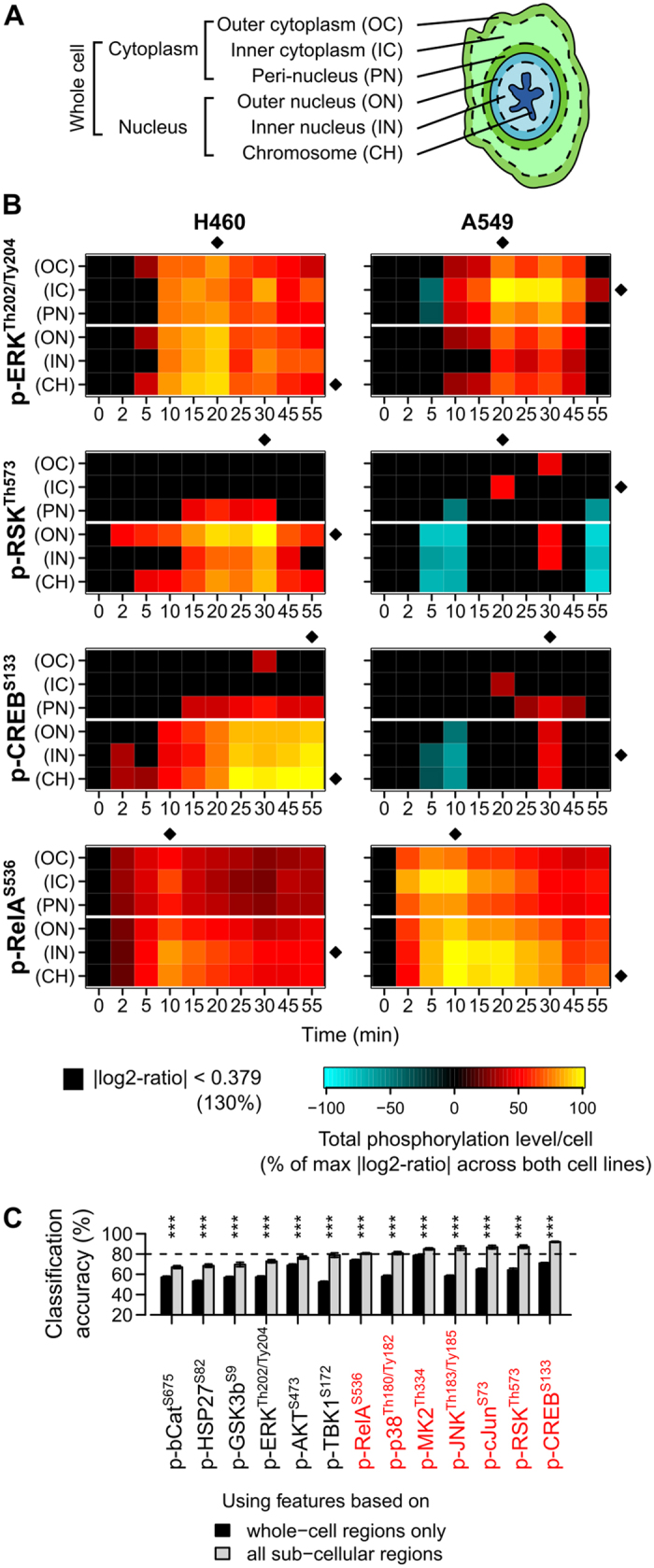
Most signals peak at different subcellular regions in sensitive and resistant cells. (**A**) Schematic showing the nine automatically detected subcellular regions. The whole-cell, cytoplasmic, and nuclear regions are composed of other subcellular regions. Please also refer to Supplementary Fig. S6 for examples of actual microscopy images depicting these regions. (**B**) Heatmaps showing changes in four of the measured signals at different subcellular regions in H460 and A549 cells treated with 300 ng/mL of TNFα. All values are log_2_ ratios to the time-zero values (without TNFα treatment). All changes <30% are colored in black. For visualization only, the log_2_ ratios for each signal are divided by their maximum absolute value across both cell lines in all regions. (Diamonds = subcellular regions or time points with the maximum levels). (**C**) Mean balanced accuracies in classifying H460 and A549 cells using support vector machines based on the phosphorylation events of individual signals (dashed lines = selection threshold at 80%, red = signals selected for the second stage; ***P < 0.001, two-sided t-test; error bars = standard deviations). The values were estimated using three-fold cross validations with three random fold divisions.

To further investigate these 13 candidate signals at the subcellular level, we quantified their total phosphorylation levels in nine different subcellular regions using automated image analysis (Fig. 3A and Supplementary Fig. S6). A total of 1170 phosphorylation events per cell line were measured (Fig. 3B and Supplementary Fig. S7). These high-resolution spatiotemporal measurements revealed several interesting trends that were not obvious from whole-cell measurements.

First, we found that most signals peaked at different subcellular regions in A549 and H460 cells. For example, the phosphorylation levels of ERK at threonine 202/tyrosine 204 (p-ERK^Th202/Ty204^), p90 ribosomal s6 kinase at threonine 573 (p-RSK^Th573^), cJun terminal kinase at threonine 183/tyrosine 185 (p-JNK^Th183/Ty185^) and cJun at serine 73 (p-cJun^S73^) peaked in either the nuclear regions of H460 cells or the cytoplasmic regions of A549 cells (Fig. 3B and Supplementary Fig. S7). Our results suggest that the transduction of these signals might be partially blocked in the more resistant A549 cells.

Second, we found that some signals exhibited multiphasic responses that peaked in different subcellular regions at different times. For example, in both A549 and H460 cells, the first phase of p-RelA^S536^ occurred in the inner cytoplasmic region at ~5–10 min (Fig. 3B). However, in A549 cells, the second phase of p-RelA^S536^ was stronger and eventually peaked in the chromosomal regions at ~10–20 min; but in H460 cells, it was weaker and only peaked in the inner nuclear region (Fig. 3B). These observations agree with a previous non-imaging-based study, which found that TNFα-induced phosphorylation of RelA on Ser536 first occurs at the cytoplasm, and then leads to the nuclear translocation of NF-κB^24^. NF-κB is known to regulate the expression of many pro-survival genes and suppress cell death^25^. However, nuclear translocation of NF-κB alone is insufficient to initiate the transcription of these genes. The process is also controlled by a number of additional post-translational modifications (such as phosphorylation, methylation, and acetylation) and associations with other co-factors in the nucleus^26^,^27^. Although we found that p-RelA increased in the nuclear regions of H460 cells, its lack of strong co-localization with the chromosomal regions (Fig. 3B) suggests that H460 may be lacking some of these additional mechanisms, and thus unable to fully activate NFκB’s transcriptional activity. This may contribute to H460’s sensitivity to TNFα.

Third, we found that known kinase-substrate pairs tended to have correlated and sometimes delayed temporal phosphorylation patterns (for example, p-ERK^Th202/Ty204^, p-RSK^Th573^, and p-CREB^S133^ in the nuclear regions of H460 cells, Fig. 3B). Taken together, these observations demonstrate that signaling measurements based on subcellular regions may provide more discriminative information than measurements based on whole-cell regions.

### Spatial information increases classification accuracy

To study each signal individually, we divided all the 1170 phosphorylation events into 13 groups according to their associated signals. Then, we used a machine learning algorithm called support vector machine (SVM)^28^,^29^ to classify individual H460 and A549 cells into two distinct categories, “sensitive” or “resistant”, based on each of these groups of phosphorylation events independently (Methods). We used a radial-basis-function kernel^29^ for the SVM algorithm, and a 3-fold cross validation procedure^30^ to evaluate the binary classifiers. For each group, the result was a classification accuracy score, balanced accuracy, that we used as an indicator of the ability of the signal associated with the group to distinguish between TNFα-sensitive and -resistant cells. Since cells from the same wells may be randomly selected for different cross validation folds, there may be a bias in our estimated classification accuracy scores. However, the exact same procedure was used to analyze all the signaling event groups, and thus the bias was equally applied to all the groups and should have a negligible effect in the relative ranking of the groups. Overall, we found that phosphorylation events based on subcellular regions gave higher balanced accuracy values than those based on whole-cell regions, indicating the importance of measuring spatial information (Fig. 3C). As expected, all three transcription factors (RelA, cJun, and CREB) produced classifiers with high balanced accuracy values. Interestingly, after considering spatial information, four intermediate kinases, namely p38, mitogen activated protein kinase activated protein kinase 2 (MK2), RSK, and JNK, also produced classifiers with higher accuracy than RelA. Among them, JNK and MK2 were also previously identified to be highly predictive of TNFα-induced apoptosis in a colon cancer cell line^1^. We also obtained similar top rankings of signals using a linear kernel for the SVM, or a 10-fold cross validation procedure (Supplementary Fig. S8). Based on these results, we selected seven candidate signals with the highest balanced accuracy for further testing (Fig. 3C).

### p-RSK^Th573^ is the most discriminative signal

In the next stage of our search, we stained and imaged the seven selected signals in all cell lines (Supplementary Data S1). For H460 and A549 cell lines, we found that the regenerated signaling responses were highly reproducible, again demonstrating the reliability of our methods (Supplementary Fig. S9). For each signal, we modeled the relationship between all its phosphorylation events and the cytotoxic sensitivity index using a linear regression model. This method allows us to generate continuous estimates of sensitivity levels, and avoid assigning cell lines into distinct “sensitive” or “resistant” categories. Furthermore, the relative importance of a phosphorylation event can be easily determined from the magnitude of the weight parameter (*β*_*i*_) associated to the event in our models (Methods). We determined the optimal values for these weight parameters using an elastic net algorithm combined with a bootstrapping procedure^7^,^15^.

**Figure 4:**
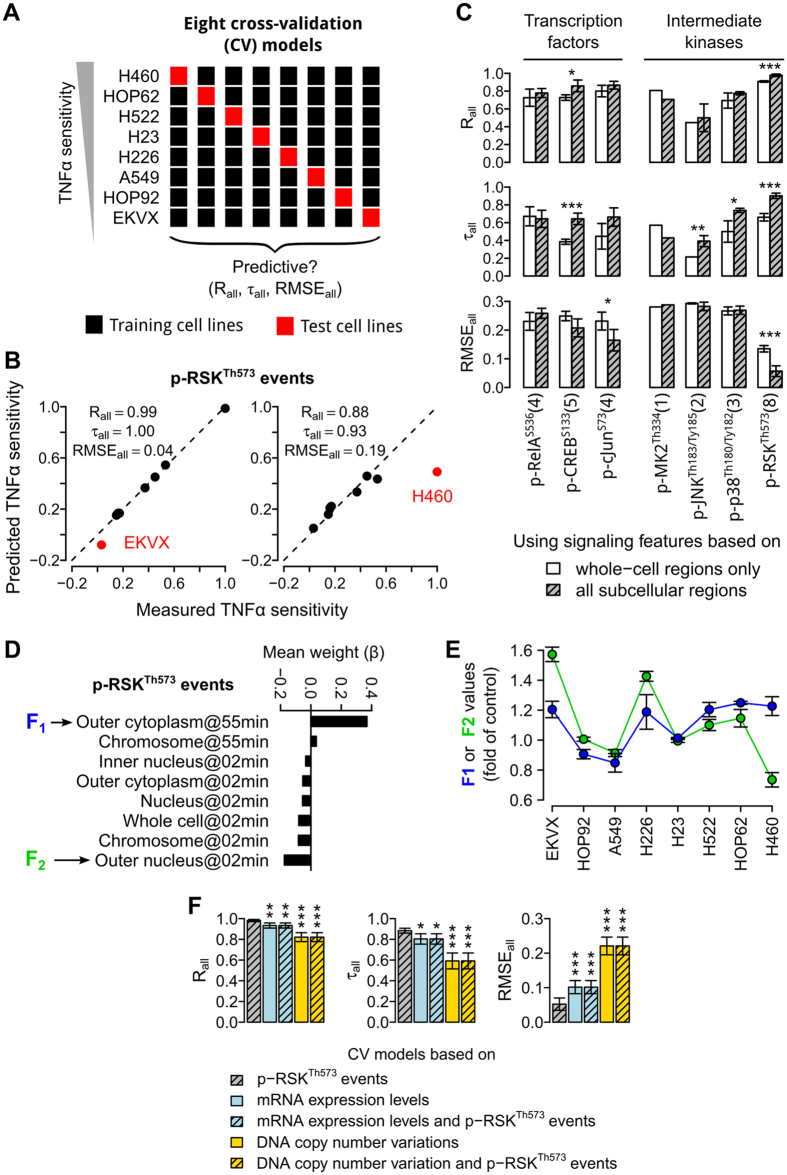
p-RSK^Th573^ is the most discriminative signal. (**A**) Schematic showing the cross validation (CV) procedure used to train and test our linear regression models. For each signal, up to eight CV models were trained and tested. Pearson’s correlation coefficient (R_all_), Kendalls’ correlation coefficient (τ_all_), and root mean squared error (RMSE_all_) were used to evaluate the models. (**B**) Scatter plots showing the measured versus predicted sensitivity levels of NSCLC cells (dots) for two of the CV models based on the p-RSK^Th573^ (black = training, red = test cell lines). In these two CV models shown, the most sensitive (H460) or resistant (EKVX) cells were not used for model training, respectively. (**C**) Mean performance values of the CV models based on each or combinations of the seven selected signals. For each condition, the number of CV models that could be fitted is shown in the parentheses (*P < 0.05, **P < 0.01, ***P < 0.001, one-sided t-test.) (**D**) The average weights (*β*_*i*_) of p-RSK^Th573^ events across the eight CV models. Only weights with |*β*_*i*_| > 0.03 are shown. F_1_ and F_2_ = two p-RSK^Th573^ events with the highest |*β*_*i*_| values. (**E**) The average values of F_1_ (blue) and F_2_ (green) in all the eight cell lines (error bars = SEM). (**F**) Mean performance values of the CV models based on p-RSK^Th573^ events, genome-wide mRNA expression levels, DNA copy number variations, or combinations of these features of the cell lines (*P < 0.05, **P < 0.01, ***P < 0.001, one-sided t-test with respect to the CV model based on p-RSK^Th573^ events).

We rigorously tested each individual signal using a cross validation (CV) procedure^30^, where each of the eight cell lines was used in turn to test the regression model trained based on other seven cell lines (Fig. 4A and B). For each signal, up to eight CV models were trained and tested, because the signal may not be correlated to TNFα sensitivity, and thus not all training cell sets could be optimally fitted with linear regression models. The final prediction accuracy value for the signal was estimated by averaging the values obtained only from the CV models that converged. Training cell sets that did not converge were not included. We found that an intermediate kinase, RSK, produced converged regression models in all of the eight training cell sets (Fig. 4C). Models based on transcription factors, namely RelA, c-Jun, and CREB, converged in 4, 4, and 5 of the training cell sets; while models based on other kinases, namely MK2, JNK, and p38, converged in 1, 2, and 3 of the training cell sets. Signals with fewer converged models usually had lower prediction accuracy values (Fig. 4C). Among all the signals, p-RSK^Th573^ had the highest prediction accuracies (Pearson’s correlation coefficient R_all_ = 0.98, Kendall’s correlation coefficient τ_all_ = 0.88, and root mean square error RMSE_all_ = 0.05; Fig. 4C). The signal could still give reasonable accuracies when cell lines with extreme sensitivity levels were not used for model training (Fig. 4B). We also found that inclusion of spatial information significantly increased the prediction accuracies of p-RSK^Th573^ (P < 0.001, one sided t-test; Fig. 4C).

Interestingly, when averaged across all the CV models, two p-RSK^Th573^ events stood out: its total levels in the outer cytoplasmic region at 55 min (represented by “F_1_” and with *β* = 0.369) and the outer nuclear region at 2 min (represented by “F_2_” and with *β* = −0.175, Fig. 4D and Supplementary Fig. S10). The sign of the coefficients indicates that F_1_ is positively correlated to TNFα sensitivity, while F_2_ is negatively correlated to TNFα sensitivity. We compared the values of these two features in all eight cell lines, and found that F_2_ tends to have higher values than F_1_ in the resistant cells, irrespective of the absolute levels of F_1_ and F_2_ (Fig. 4E and Supplementary Fig. S10). In fact, both the resistant EKVX and H226 cells have strong activations of p-RSK^Th573^ (Fig. 4E and Supplementary Fig. S10). Therefore, the combination of these two features may reflect the temporal difference in p-RSK^Th573^ activation dynamics between sensitive and resistant cells, where the resistant cells tend to have earlier activation at the outer-nuclear region and less sustained activation at the outer-cytoplasmic region than the sensitive cells.

### Signaling features are more accurate and robust than transcriptomic features

Quantitative models based on basal genomic and/or transcriptomic features have been used to predict cytotoxic sensitivity of cancer cell lines^7^,^8^. We wondered to what extent genomic and transcriptomic features can predict the TNFα sensitivity levels of our NSCLC cell lines. To perform a systematic comparison, we obtained genome-wide measurements of mRNA expression levels and DNA copy number variations for our cell lines from the NCI CellMiner website^31^. We found that the accuracies of regression models based on genomic or transcriptomic features are significantly lower than the p-RSK^Th573^ model (P < 0.01, one-sided t-test, Fig. 4F). This may be partly due to the lack of TNFα-response measurements in these basal models, and stimulated (signaling or transcriptomic) models are likely to be more informative and accurate than basal models. We also determined the accuracies of regression models based on both genomic/transcriptomic features and RSK events, but found that the addition of RSK events does not improve the performance of these models (Fig. 4F). This is likely due to the inability of elastic net, which is a state-of-the-art algorithm used in several recent studies^7^,^15^, in selecting discriminative features from high dimensional data. Furthermore, we found that the two p-RSK^Th573^ features with the highest weights were consistently detected in seven or eight of the eight CV models, respectively; whereas most of the selected transcriptomic features (~92%) were detected in only one of the eight CV models (Supplementary Fig. S11). These results show that there may be large numbers of gene expression profiles that are highly correlated to each other and TNFα sensitivity, and thus arbitrary subsets were selected in a particular CV fold. Another possibility is that many genes are correlated to TNFα sensitivity, but only in specific subsets of our cell lines. This problem may be alleviated by increasing the number of training cell lines, but similar disconcordance was also observed in the models generated in two recent large-scale studies that used hundreds of cancer cell lines^32^. Our results suggest that signaling features provide important information that is complementary to basal genomic/transcriptomic features in predicting cytotoxic sensitivity.

### RSKs attenuate TNFα-induced cytotoxicity

**Figure 5:**
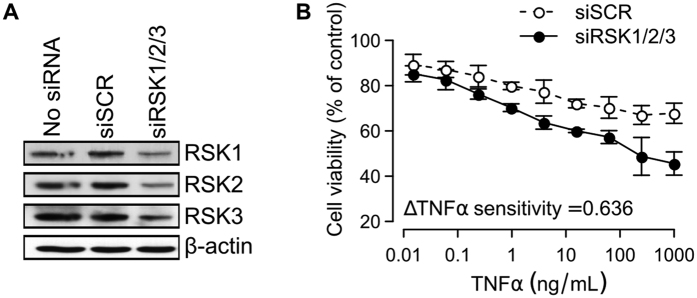
RSKs attenuate TNFα response. (**A**) Western blots showing the protein expression levels of RSK1, 2, and 3 in untreated (no siRNA), scrambled (siSCR) and RSK1/2/3 (siRSK1/2/3)-siRNA treated H460 cells. (**B**) Dose response curves showing the percentages of viable H460 cells after 24 h of TNFα treatments (n = 3, error bars = standard errors of the means, ΔTNFα sensitivity = normalized TNFα sensitivity index with respect to siSCR).

RSKs are a family of protein kinases that are more commonly known to be activated by receptor tyrosine kinases under growth factor stimulations^33^. However, our results suggest that RSKs may also be involved in TNFα-induced cell death. To further determine the role of RSKs, we used small-interfering RNA (siRNA) to reduce the expression levels of all three isoforms of RSK, namely RSK1, 2, and 3, in H460 cells (Fig. 5A). We found that siRSK1/2/3 alone (without TNFα) reduced the number of viable cells, and increased the variability of immunofluorescence staining experiments. To overcome this, we switched to a resazurin-based cell viability assay, which is less sensitive to cell detachment, and increased the measurement time to 24 hours. This cell viability assay was found to generate TNFα sensitivity measurements that are consistent to our earlier immunofluorescence-based cell death assay (Supplementary Fig. S12). We found that knockdown of RSKs further sensitizes H460 cells to TNFα (Fig. 5B). Our results also agree with a previous finding that RSK2 directly phosphorylates IκBα, lending to the activation of NF-κB^34^. Taken together, these results suggest that RSKs attenuate TNFα-induced cytotoxicity.

### The final RSK model predicts the effects of new co-treatments

**Figure 6:**
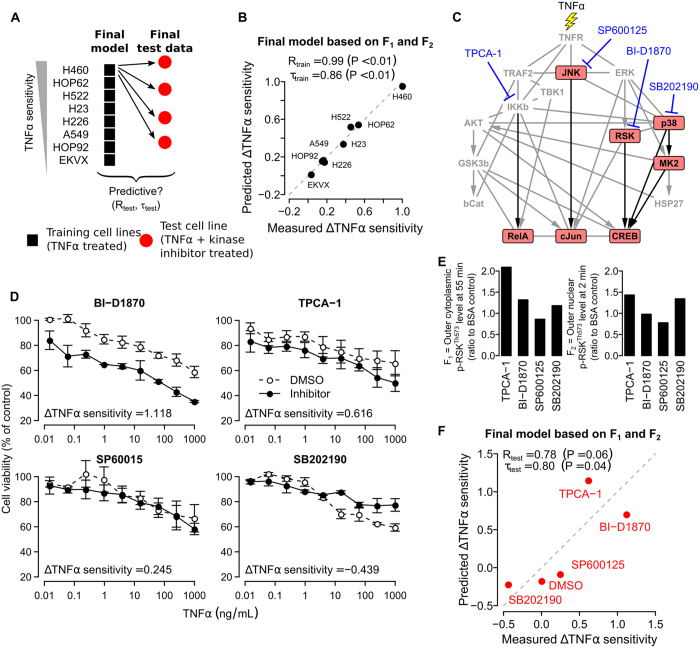
A linear regression model based on two RSK events predicts the effects of new chemical co-treatments. (**A**) Schematic showing the procedure used to train a final model using all the cell lines, and test the model in H460 cells treated with TNFα and four different kinase inhibitors. (**B**) Scatter plots showing the measured versus predicted sensitivity levels of NSCLC cells (dots) for the final model based on F_1_ and F_2_, which was trained on all the cell lines (black = training cell lines). (**C**) Schematic showing the main targets of the four selected small-molecule kinase inhibitors (red node = the seven signaling proteins selected during the first stage of our heuristic search, black edges = known direct phosphorylation interactions involving the selected signaling proteins, gray edges = other known functional associations, physical interactions, or phosphorylation interactions). (**D**) Dose response curves showing the percentages of viable H460 cells after pre-treatment of DMSO control (0.1%), TPCA-1, BI-D1870, SB200190, or SP600125 (all 10 μM) for one hour and then treatment of 300 ng/mL TNFα for 24 hours (n = 2 or 3, error bars = standard errors of the means, ΔTNFα sensitivity = normalized TNFα sensitivity index with respect to DMSO). (**E**) Average values of F_1_ and F_2_ quantified from the immunofluorescence images of H460 cells treated with TNFα and the inhibitors (Supplementary Fig. S13). (**F**) Scatter plots showing the measured versus predicted ΔTNFα sensitivity indices of H460 cells based on the final p-RSK^Th573^ model (dashed line = diagonal line, red = new test data that was not used to train the model, R_test_ = Pearson’s correlation coefficient, τ_test_ = Kendalls’ correlation coefficient, P-values shown were obtained from significance tests that the correlations are larger than zero).

We hypothesize that two p-RSK^Th573^ features with the highest weights (F_1_ and F_2_) are sufficient to predict the combined effects of TNFα and other co-treatments, even though these co-treatments may not directly affect RSK and may have multiple intracellular targets. To test our hypothesis, we trained a final linear regression model based on the values of these two features in all eight cell lines (Fig. 6A). We found that the final model can almost perfectly fit the responses of all the cell lines, including H460 and EKVX (Fig. 6B). Then, we applied the final model without any further training or modification to a new set of p-RSK^Th573^ measurements collected from H460 cells co-treated with different small-molecule kinase inhibitors. Based on the results from the first stage of our heuristic search, the NF-κB, JNK, RSK and p38 signaling pathways showed differential activations between sensitive and resistant cells (Fig. 3C). Therefore, as a proof of concept, we chose four small-molecule inhibitors, namely TPCA-1 to block the NF-κB, SP600125 to block the JNK, BI-D1870^35^ to block the RSK, and SB202190 to block the p38 signaling pathways (Fig. 6C). These inhibitors were also chosen because they are known to have other non-specific intracellular targets^36^,^37^,^38^,^39^. They provide us with a simplistic but realistic scenario of a screening experiment for small molecules that can modulate cellular sensitivity to a cytotoxic agent.

We performed the same resazurin-based cell viability assay on H460 cells co-treated with these inhibitors and TNFα. For each inhibitor, we computed a TNFα sensitivity index by measuring the area under its dose response curve (Fig. 6D). Since we were more interested in the change in TNFα sensitivity induced by an inhibitor, we normalized the obtained sensitivity index by the sensitivity index of the solvent control, DMSO. We found that three of the inhibitors clearly changed the TNFα sensitivity levels of H460 cells. In particular, SB202190 de-sensitized, while TPCA-1 and BI-D1870 sensitized the cells to TNFα (Fig. 6D). SP60015 was found to have mild or no effect on TNFα sensitivity. Our results agree with the known roles of p38^40^ or NF-κB^22^ in promoting or suppressing TNFα-induced cell death, respectively. Interestingly, the BI-D1870 results also agree to our earlier siRNA results (Fig. 5B), and further confirm a role of RSKs in TNFα response. Together, these four compounds and the DMSO solvent provided a set of relevant perturbations for us to test our final RSK model.

We stained and imaged the p-RSK^Th573^ subcellular localization patterns in H460 cells pre-treated with these four inhibitors for an hour, and then co-treated with TNFα (Fig. 6E and Supplementary Fig. S13). At the basal untreated condition, we found that most H460 cells had very low levels of p-RSK^Th573^, except for a small subpopulation of mitotic cells that could be easily characterized by their condensed chromosomes (Supplementary Fig. S13). In DMSO-treated cells, TNFα induced rapid nuclear (especially near the outer nuclear regions) phosphorylation of RSK^Th573^ starting from 2 min or earlier (Fig. 3B and Supplementary Fig. S13). However, the response was highly heterogeneous and not all cells were activated at the same time. In SB202190-treated cells, TNFα induced a stronger response, especially at 2 min (Fig. 6E and Supplementary Fig. S13). This agrees with the negative correlation between F_2_ and TNFα sensitivity in our regression model (Fig. 4D). In TPCA-1-treated cells, TNFα also induced a stronger response. However, we found an increase in the cytoplasmic level of p-RSK^Th573^ at 55 min (Fig. 6E and Supplementary Fig. S13). Again, this agrees with the positive correlation between F_1_ and TNFα sensitivity in our regression model (Fig. 4D).

After quantifying the values of F_1_ and F_2_ for all the inhibitors, we applied our final model to these values, and found that the predicted and measured changes in TNFα sensitivity were positively correlated (Pearson’s correlation coefficient R_test_ = 0.78, Kendall’s correlation coefficient τ_test_ = 0.80, Fig. 6F). Importantly, our model could accurately predict the sensitization effects of TPCA-1 and BI-D1870. We also repeated the same final model training and testing procedure but using two features measuring total RSK^Th573^ levels at the whole-cellular regions at 2 and 55 mins. We found that this model is unable to correctly predict the effects of the inhibitors (Supplementary Fig. S14). Again, the result demonstrates the importance of spatial information in modeling TNFα response.

## Discussion

Our study provides a detailed view of the complex spatiotemporal dynamics of signaling cascades involved in the generation of differential phenotypic responses to a cytotoxic agent. We show that many intermediate protein kinases are commonly phosphorylated in sensitive and resistant cells, and therefore information does not propagate in distinct pathways. However, within one hour of treatment, transcription factors start to show differential phosphorylation levels, suggesting different transcriptional programs have been initiated. Therefore, signal divergence may occur either at or before the level of transcriptional factors in signaling cascades. In NSCLC cells treated with TNFα, we found that the spatiotemporal phosphorylation patterns of RSK are good predictors of cytotoxic sensitivity. Other earlier signaling proteins that drive RSK may also exhibit diverged responses, but we did not observe such behavior for ERK that is upstream of RSK (Fig. 3C). Our results suggest that signal divergence may start to occur at the intermediate level of signaling cascades.

We also show that simple regression models based on two spatiotemporal features of p-RSK^Th573^ can predict the eventual phenotypic outcomes. This suggests that intermediate signaling proteins at or near the divergence points of a signal transduction cascade may be used to predict the effects of external perturbations to the network, even though the perturbations may not directly target these signaling proteins. Consequently, a complex signaling network may be represented by compact, spatiotemporal signatures of these signaling proteins, which are less likely to overfit and thus more preferable than complex models with large numbers of variables^41^.

The high prediction accuracy of our signaling models is mainly due to the spatiotemporal features of signaling responses. These subcellular features may reflect the different biological functions performed by the proteins at different subcellular regions^42^,^43^. Furthermore, subcellular features are usually more robust than whole-cell features, because linear combinations of subcellular features may cancel out experimental noise that is common to the measurements at different regions. Irrespective of the underlying reasons, our study has shown that the removals of subcellular-region-specific features dramatically reduce the performance of our models (Figs 3C and 4C), and make it harder to identify diverged signals. Therefore, inclusion of spatiotemporal features is critical for future constructions of signaling models.

In our study of TNFα response, we show that early changes (within one hour of treatment) in intermediate signals are sufficient to accurately predict the eventual phenotypic effects of TNFα and co-treatments. Therefore, our study provides a general framework for the development of rapid, signaling-based cytotoxicity screens that do not require *in vitro* propagation of cells. Our signaling profiling and modeling methods are general and may be directly applied to other cytotoxic agents or perturbations, such as cytokines, drugs, toxicants, radiation, or mechanical stress, that induce rapid signaling responses. Many primary or *ex vivo* samples of human cells grow slowly or cannot be propagated under *in vitro* conditions, and therefore direct assessments of cytotoxicity sensitivity of these cells remain challenging^44^. In principle, our signaling imaging and profiling methods can be directly applied to these cells and screen for agents that are more toxic to specific cells or individuals, or co-treatments that may sensitize or desensitize cells to these effects. Such screenings may be applicable to the development of new precession and/or combinatorial medicines, or the assessment of the toxicity of chemical compounds.

## Materials and Methods

### Cell culture and treatment

We obtained frozen NSCLC cell lines, EKVX, HOP92, A549, H522, HOP62, H226, H23 and H460, as part of the NCI-60 cell line panel from NCI-Frederick. Only one NSCLC cell line, H322M, from the panel was excluded because it does not form monolayers typical of epithelial cells in culture. All cell lines were grown in RPMI medium with 10% fetal bovine serum (FBS) at 37 °C and 5% CO_2._ Media were replaced every 2–3 days.

All the imaging experiments were performed in optical bottom black 384-well plates (#164586, Nunc) coated with 2 mg/ml fibronectin (#sc-29011, Santa Cruz) in PBS overnight at room temperature. First, cells were seeded (EKVX = 7000, HOP92 = 6000, A549 = 6000, H226 = 7000, H23 = 8000, H522 = 8000, HOP62 = 4000 and H460 = 6000 cells/well) and left to attach overnight. The following day, the culture media was replaced with 15 μl serum-free media and left overnight to starve the cells. Then, similar to previous studies^17^,^45^, 33 μM cyclohexamide (#01810, Sigma) was used to sensitize the cells for 1 hour and then removed from the cells. Finally, cells were treated with TNFα (#IY-223, iDNA) at either 0.01–3000 ng/mL (10 three-fold serial dilutions) for eight or twenty-four hours (cytotoxicity assays), or 300 ng/mL for 2, 5, 10, 15, 20, 25, 30, 45 or 55 min (signaling profiling assays). To examine the effect of inhibition of intracellular signaling proteins on the TNFα sensitivity of H460 cells, we added 10 μM of BI-D1870 (#15264, Cayman Chemicals), TPCA-1 (#2559, R&D Systems), SP600125 (#10010466, Cayman Chemicals), SB200190 (#10010399, Cayman Chemicals), or DMSO control (0.1%) to the cyclohexamide and BSA/TNFα solutions. Stock solutions of these chemical inhibitors were made in DMSO at 10 mM, aliquoted and stored at −20 °C. Fresh aliquots of inhibitors were utilized in each experiment to avoid the detrimental effects of freeze-thaw cycles. We performed 2 to 4 replicated experiments.

### Immunofluorescence assay

After TNFα treatment, cells were fixed with 4% paraformaldehyde for 15 min at room temperature and washed with TBS (three times 5 min). Cells were permeabilized in TBS buffer containing 0.1% triton-X-100 (TBS-T) for 5 min at room temperature and blocked for 1 hour at room temperature in 5% Bovine serum albumin (BSA) (#A7906, Sigma) in TBS. Then, cells were incubated with primary antibodies in 5% BSA-TBS overnight at 4 °C. All primary antibodies were purchased from Cell Signaling and are as the following: anti-phospho-βCatenin (#4176 S), anti-phospho-TBK1 (#5483), anti-phospho-ERK (#9106), anti-phospho-HSP27 (#2406), anti-phospho-p38 (#4511), anti-phospho-GSK3β (#5558), anti-phospho-RSK (Thr573) (#9346), anti-phospho-cJun (#3270), anti-phospho-Akt (#4060), anti-phospho-JNK (#9255), anti-phospho-CREB (#9198), anti-phospho-RelA (#3033) and anti-phospho-MK2 (#3007). After washing are re-blocking for 15 min, cells were incubated with anti-rabbit-Alexa647 and anti-mouse-Alexa546 secondary antibodies (#A-21245 and #A-11030, Invitrogen) for 1 hour at room temperature. Additionally, cells were stained with a COX-IV antibody directly conjugated with Alexa-488 dye (#4853) for 2 hours to detect mitochondria and cell boundary, and 0.5 μg/ml Hoescht 33342 (#H-1399, Invitrogen) for 1 min to detect DNA and nuclear boundary. Finally, cells were stored in TBS in sealed light protected plates at 4 °C until imaging.

### Cytotoxicity assay

The same immunofluorescence assay protocol as described above was used for the cytotoxicity assays, except for the following differences. One hour before fixation, 5 μM of 5-ethynyl-2′-deoxyuridine (EdU) was added to the wells and then detected using the Click-iT Edu Assay (#C-10350, Invitrogen). Cells were stained with anti-cleaved-Caspase3 (#9664, Cell Signaling) at 4 °C overnight, anti-rabit-Alexa546 secondary antibody (#A-11035, Invitrogen) at room temperature for an hour, and HCS Nuclear Mask blue (#H32720, Invitrogen) at room temperature for 15 min. For the siRNA and small-molecule kinase inhibitor experiments, we measured cell viability after 24 h TNFα treatment using the CellTitre Blue assay (Promega).

### siRNA knockdown and validation assays

H460 cells were seeded in 96 well plates (5000 cells/well) in 10% serum RPMI. The next day, the cells were transfected with siRNA directed against RSK1/2/3 (siGenome, Dharmacon) using DharmaFECT 1 transfection reagent (Dharmacon). After 48 hours, the cells were starved in serum free RPMI for 6 hours prior to treatment with TNFα (0–1000 ng/ml). Cell Viability was measured after 24 hours of TNFα treatment using the CellTitre Blue assay (Promega). To verify siRNA knockdown of RSK1, 2 and 3 protein, whole cell lysates were harvested at 48 hours post transfection, run on SDS PAGE gels, transferred to nitrocellulose membrane and probed using isoform specific antibodies: RSK1 (#9333), RSK2 (#9340) and RSK3 (#9343, all from Cell Signaling). Secondary antibodies used were anti-rabbit-HRP and anti-mouse-HRP (GE healthcare). Blots were stripped and re-probed using a β-actin mouse polyclonal antibody (Santa Cruz) as a loading control.

### Image acquisition and analysis

We imaged plates at 20x using an inverted fluorescence microscope (Axio Observer, Zeiss) equipped with a 12-bit charge-coupled device camera (CoolSNAP HQ2, Photometric). Nine positions were imaged per well, and the raw images were saved as 1392 × 1040 pixels, 16-bit TIFF files. We minimized non-uniform background intensities in the images using the rolling-ball algorithm implemented in ImageJ (v1.49d, NIH). We used the cellXpress software (v1.4, Bioinformatics Institute)^12^ to automatically segment individual cells based on the Hoechst and COX-IV staining patterns, identify nine different subcellular regions (whole cell, cytoplasm, nucleus, outer cytoplasm, inner cytoplasm, peri-nucleus, outer nucleus, inner nucleus, and chromosome) from each of the segmented cells, and quantify the total staining levels of the phospho-protein antibodies in each of the subcellular regions. Detail instructions for these steps and software installation packages are publicly available at http://www.cellXpress.org. The following region detection parameters were used: nuclear boundary size = 3 pixels, perinuclear boundary size = 6 pixels, and cytoplasmic boundary size = 4 pixels. To avoid potential biases, we did not use the phospho signals for cell segmentation, and instead used the COX-IV staining (Supplementary Fig. S2).

### Cytotoxicity index calculation

We quantified two cytotoxicity readouts, namely the total cleaved caspase-3 level at the whole cellular region and the total EdU level at the nuclear region, from the microscopy images of NSCLC cell lines treated with TNFα for eight hours. Three experimental replicates were performed for each cell line. To calculate the activity area under a response curve (*AUC*), we first determined the minimum response (*r*_min_) of a cell line by measuring the median readout value across the replicates at the lowest TNFα concentration (0.01 ng/mL). Then, we computed the log2-ratios of all the measured responses (*r*_i_) with respect to the minimum response 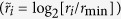, and fit a three-parameter log-logistic function to the obtained values using the drc library (v2.3.96) in the R environment. The function also estimated the confidence bands for the fitted curves shown in Fig. 1C. Finally, the fitted function was used to estimate the response values at TNFα concentrations = 10^−2^, 10^−1.5^, 10^−1^, …, 10^2.5^, 10^3^, and 10^3.5^ ng/mL, which were then summed to yield the *AUC* value for the response curve. The *AUC* values are usually positive for cleaved-caspase-3 curves, but negative for EdU curves. To derive a final cytotoxicity index (*CI*_*j*_) for each cell line *j*, we computed equation 1:


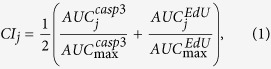


where 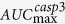 and 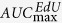 are the maximum *AUC* values across all cell lines.

For the cell viability assay, we first subtracted the average background fluorescence values estimated from blank wells from all the measured fluorescence values. Then, we calculated the percentage of viable cells by dividing each measured value with its corresponding BSA control wells. Finally, all the values were subtracted from 100% (to get the percentage of non-viable cells) and averaged to get the AUC values of the dose response curves.

### Genomic and transcriptomic datasets

We downloaded the genome-wide mRNA expression levels and DNA copy number variations of the NSCLC cell lines from the NCI CellMiner website (http://discover.nci.nih.gov/cellminer/). These genomic and transcriptomic measurements were obtained using microarrays (Human Genome CGH Microarray 44 A and 44B, Agilent Technologies; and Human Genome U133 Plus 2.0, Affymetrix, respectively) under basal conditions without TNFα treatments. Details experimental protocols for these datasets can be found from the website. The mRNA expression dataset provides the expression levels of 54675 transcripts, and the DNA copy number dataset provides the copy number variations for 42494 sequences from both coding and non-coding regions.

### Image and data processing software

We quantified 90 phosphorylation events (i.e., total intensity levels at 9 subcellular regions and 10 time points) per signal from at least two experimental replicates using the cellXpress software (v1.4, Bioinformatics Institute, http://www.cellXpress.org). These measured values were averaged across replicates, and each time series from the same subcellular regions was divided by its first value at time 0. All subsequent data analyses were performed using the R statistical environment (v3.1.0, R foundation).

### Cell classification based on support vector machines

To classify H460 and A549 cells based on an intracellular signal using support vector machines (SVM), we measured the total phosphorylation levels of the signal either in the whole-cell region or nine subcellular regions. The measurement time point was also included as a feature. Therefore, the final number of features was either two (whole-cell) or ten (subcellular regions). Due to the large number of cells, we randomly sampled 100 cells per time point. For both cell lines, we obtained a total of 4000 cells (100 cells × 10 time points × 2 cell lines × 2 replicates). We did not perform feature removal because the number of cells (samples) was much larger than the number of features. We used SVM classifiers based on either a radial basis function kernel or a linear kernel as implemented in the e1071 (v1.6-3) library. A three- or ten-fold cross validation (CV) procedure^30^ was used to estimate the average balanced accuracy in classifying the dataset into two classes (“H460” or “A549”). Before classification, all features were linearly scaled to the same range [−1, 1]. During each CV fold, the training dataset was used to determine the feature scaling coefficients, and the optimum SVM parameters (*C* and *γ*) using a grid search (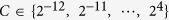 and 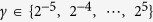). The whole CV procedure was repeated three times with random fold divisions. The final reported classification accuracies were averaged across all CV folds and random trials.

### Linear regression models

Our goal was to compare the predictivity of different types of cellular features, namely signaling, genomic and transcriptomic features. To ensure that the measured difference in performance is not due to the differences in the computational procedures used, we used the same procedures to construct linear regression models based on these three different data types. Since significant prior work has been done to model mRNA profiles^7^ or whole-cell signaling profiles^6^, we closely followed the feature filtering, linear regression modeling, and prediction performance evaluation procedures used by these previous studies. Detail descriptions of these methods can be found in the respective reports of these studies^6^,^7^. Here, we only provide a brief description of the key steps involved. Before model training, we performed feature removal and scaling using statistics measured only from the training cell lines. First, features with low variations (coefficient of variation <2%) and low correlations to TNFα sensitivity (|Pearson’s correlation coefficient| < 0.1) were removed. The removal of features with close to zero correlations was proposed by Barretina *et al*.^7^, and meant to improve the computational efficiency when the number of features is huge. Then, the values for each retained feature were converted into z-scores by subtracting the mean of the feature from the values and dividing the results by the standard deviation of the feature. We modeled the relationships between all the *n* retained features, 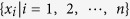, and TNFα sensitivity, *s*, using a linear regression model (equation 2):


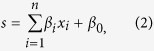


where *β*_*i*_ is a weight assigned to the *i*-th feature, and *β*_0_ is a bias term. We determined the values of all the weights using an elastic net algorithm^15^ combined with a bootstrapping procedure^7^,^46^. We used the glmnet library (v1.9–8) under the R statistical environment. The elastic net algorithm is designed to construct linear regression models when the number of features is much larger than the number of samples (or cell lines in our case). The algorithm uses two regularized regression penalty terms to balance between getting a parsimonious model (L1 term) and groups of correlated features (L2 term) as in equation 3:


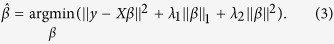


When *λ*_1_ = 0, the elastic net method is equivalent to the ridge method; when *λ*_2_ = 0, it is equivalent to the lasso method. By optimizing *λ*_1_ and *λ*_2_, we implicitly compared and optimized for different combinations of lasso and ridge methods in our analysis. In the glmnet implementation, these two terms are controlled by two parameters: *α* that controls the relative strength of the L1 and L2 terms, and *λ* that controls the overall strength of the regularized regression penalty. In a leave-one-out (LOO) CV procedure, we divided all the data into eight folds (each fold is a cell line), seven of which were used for model training. To determine an optimal *α* values from 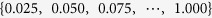 that minimized root mean squared errors, we performed another seven-fold cross validations using these seven training cell lines. For each given *α* value, the cv.glmnet function in the glmnet library automatically generates a sequence of *λ* values and searches for the corresponding optimal *λ* value. Once the optimal values for both parameters were determined, they are used to train a model using all the training cell lines. Then, a bootstrap procedure^7^,^46^ was used to generate 500 resampled datasets from the training data, determine the regression coefficients for each bootstrap dataset, and compute the occurrence of non-zero coefficients for each feature. A detail description of this procedure can be found in Barretina, *et al*.^7^ and Chen *et al*.^46^. Bootstrapped elastic nets were shown to have dramatically higher precision but select much lower numbers of features than standard elastic nets^46^. Following Barretina, *et al*.^7^, we removed features with a robustness score <0.8. After feature removal, a final model was trained based on the reduced feature set with the training cell lines, and applied to the test cell lines. The performance criteria (Pearson’s correlation coefficient - R, Kendall’s correlation coefficient - τ, and Root mean squared error - RMSE) were computed either using all the cell lines (R_all_, τ_all_, and RMSE_all_) or only the test cell lines (R_test_ and τ_test_). These three or other similar criteria were previously used to evaluate elastic net and other regression models^6^,^7^,^15^,^47^,^48^. We used a cross-validation t-test to compare the performances of different regression models^49^.

## Additional Information

**How to cite this article:** Loo, L.-H. *et al*. Early spatiotemporal-specific changes in intermediate signals are predictive of cytotoxic sensitivity to TNFα and co-treatments. *Sci. Rep.* **7**, 43541; doi: 10.1038/srep43541 (2017).

**Publisher's note:** Springer Nature remains neutral with regard to jurisdictional claims in published maps and institutional affiliations.

## Supplementary Material

Supplementary Figures and Tables

Supplementary Data S1
