# Supplementary material for: Early spatiotemporal-specific changes in intermediate signals are predictive of cytotoxic sensitivity to TNFα and co-treatments
Source: Sci Rep. 2017 Mar 8;7:43541. doi: 10.1038/srep43541 (PMC5341104; doi:10.1038/srep43541)
Supplement: Supplementary Figures and Tables [file srep43541-s1.pdf]

Supplementary Materials for

Early spatiotemporal-specific changes in intermediate signals are predictive of cytotoxic sensitivity to TNFα and co-treatments

Lit-Hsin Loo*, Nicola Michelle Bougen-Zhukov, Wei-Ling Cecilia Tan

Bioinformatics Institute, Agency for Science, Technology and Research, 30 Biopolis Street, #07-01 Matrix, Singapore 138671, Singapore

* Correspondence should be addressed to LHL: loolh@bii.a-star.edu.sg

Supplementary Figure S1-14, Table S1-3

Supplementary Materials

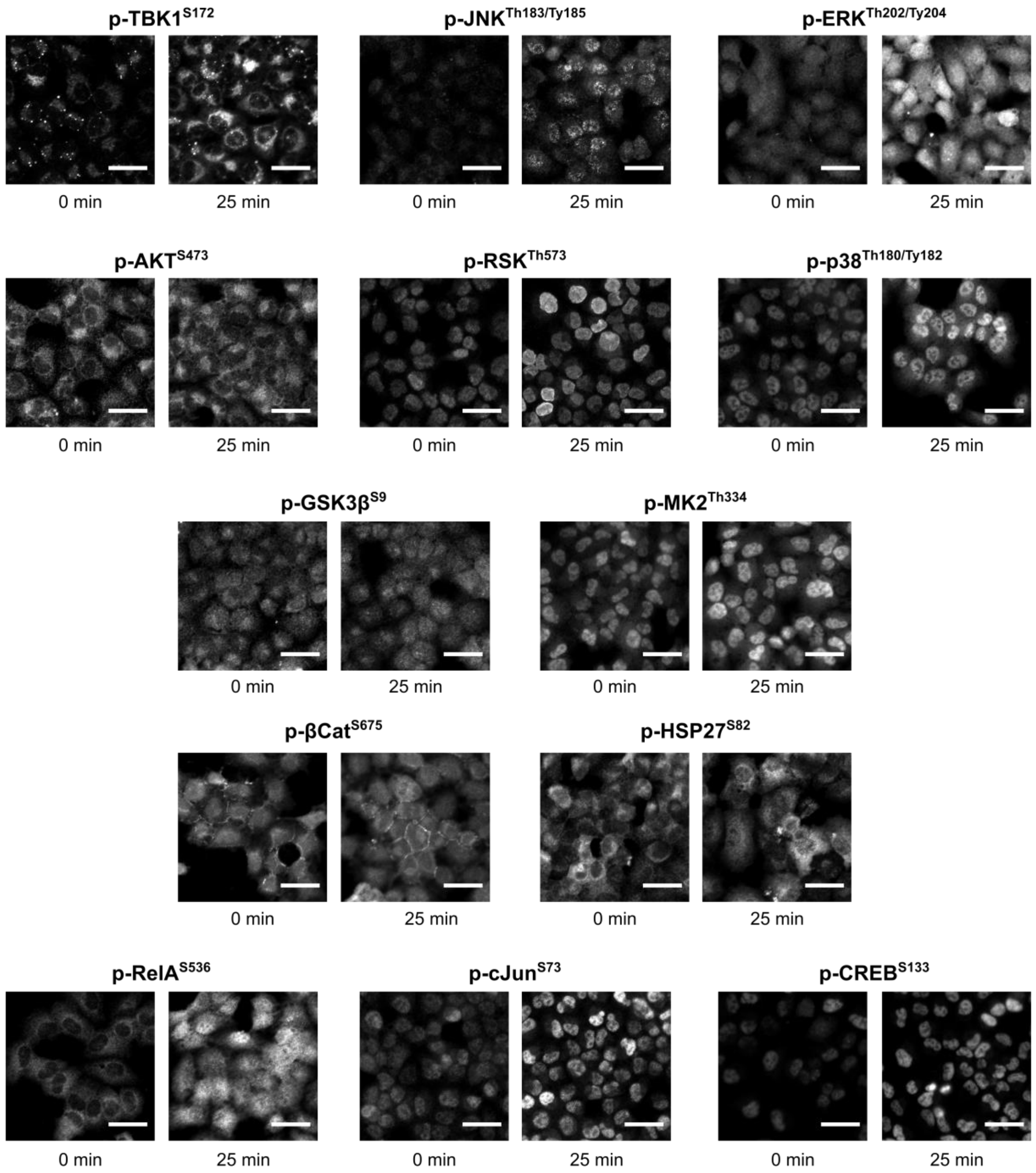

Figure S1. Examples of representative immunofluorescence images showing the staining of 13 intracellular signals in H460 cell lines treated with 0 or 25 min of TNFα. For each signal, the intensities of the images shown are scaled to the same display ranges (scale bars = 40 μm).

Supplementary Materials

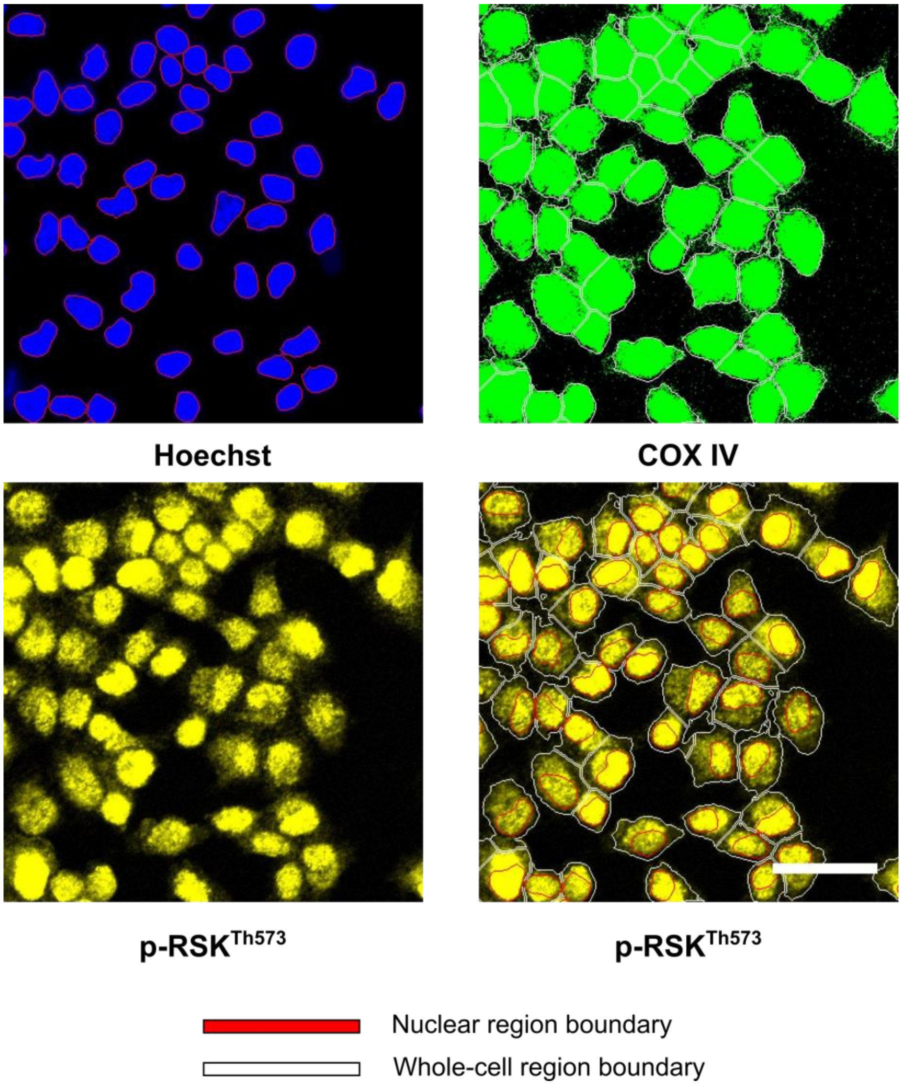

Figure S2. Examples of representative immunofluorescence images showing the automatically detected nuclear (red lines) and whole-cell (white lines) boundaries (scale bar = 40 μm). The whole-cell and nuclear regions were detected based on the COX-IV (green) and Hoechst (blue) staining, respectively. To avoid bias, the phospho-protein staining (yellow) was not used for segmentation.

Supplementary Materials

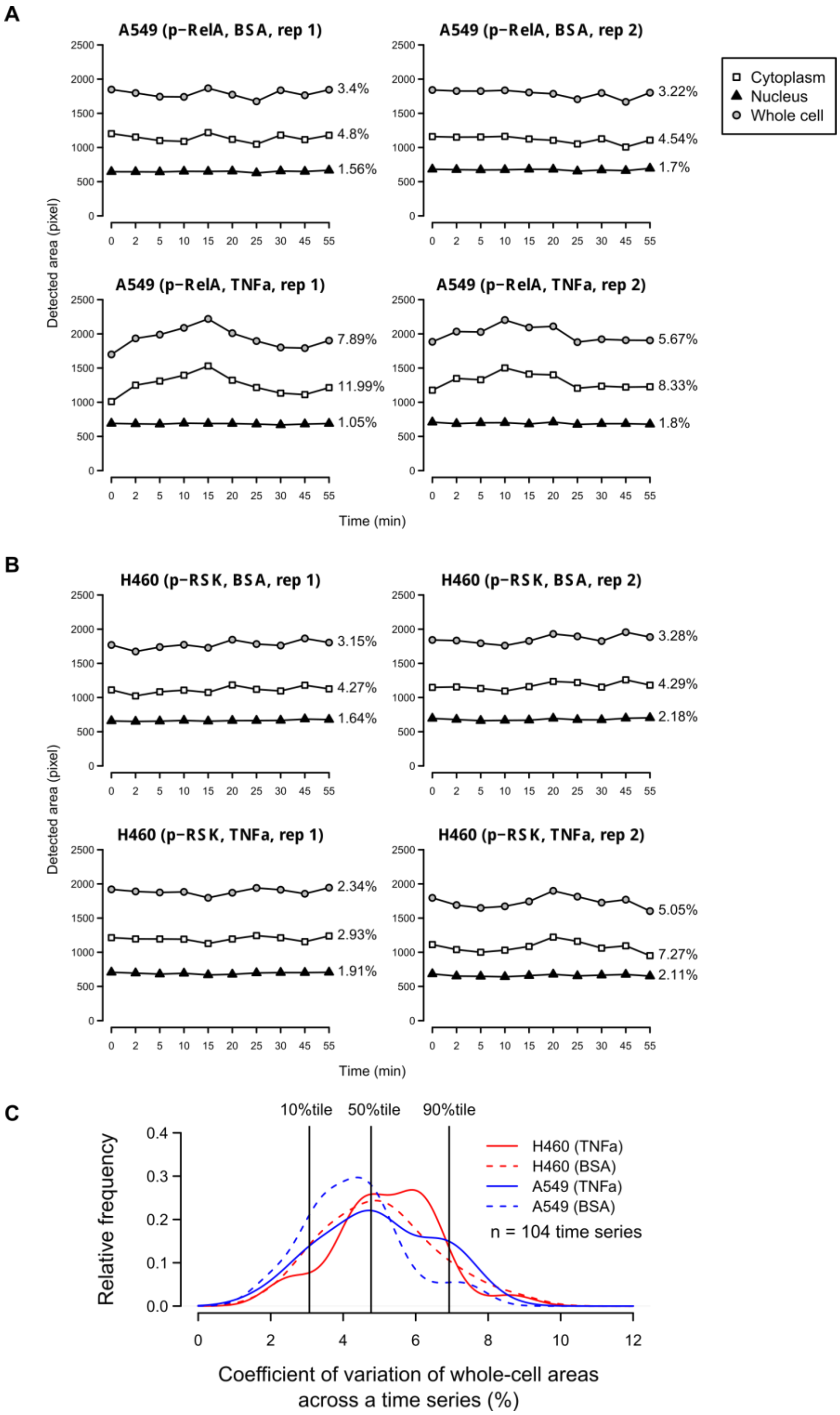

Figure S3. The areas of the automatically detected cytoplasmic, nuclear, and whole-cell regions of **A)** A549 cells stained with p-RelA^{S536}, or **B)** H460 cells stained with p-RSK^{Th573} across different time points (or wells) in our assays. The coefficient of variation (CV) of each time series is shown next to the series, rep = replicate. **C)** Probability distribution functions of the CV values for all the time series of A549 (blue) or H460 (red) cells. (Vertical black lines = percentiles of the CV values across all the time series).

Supplementary Materials

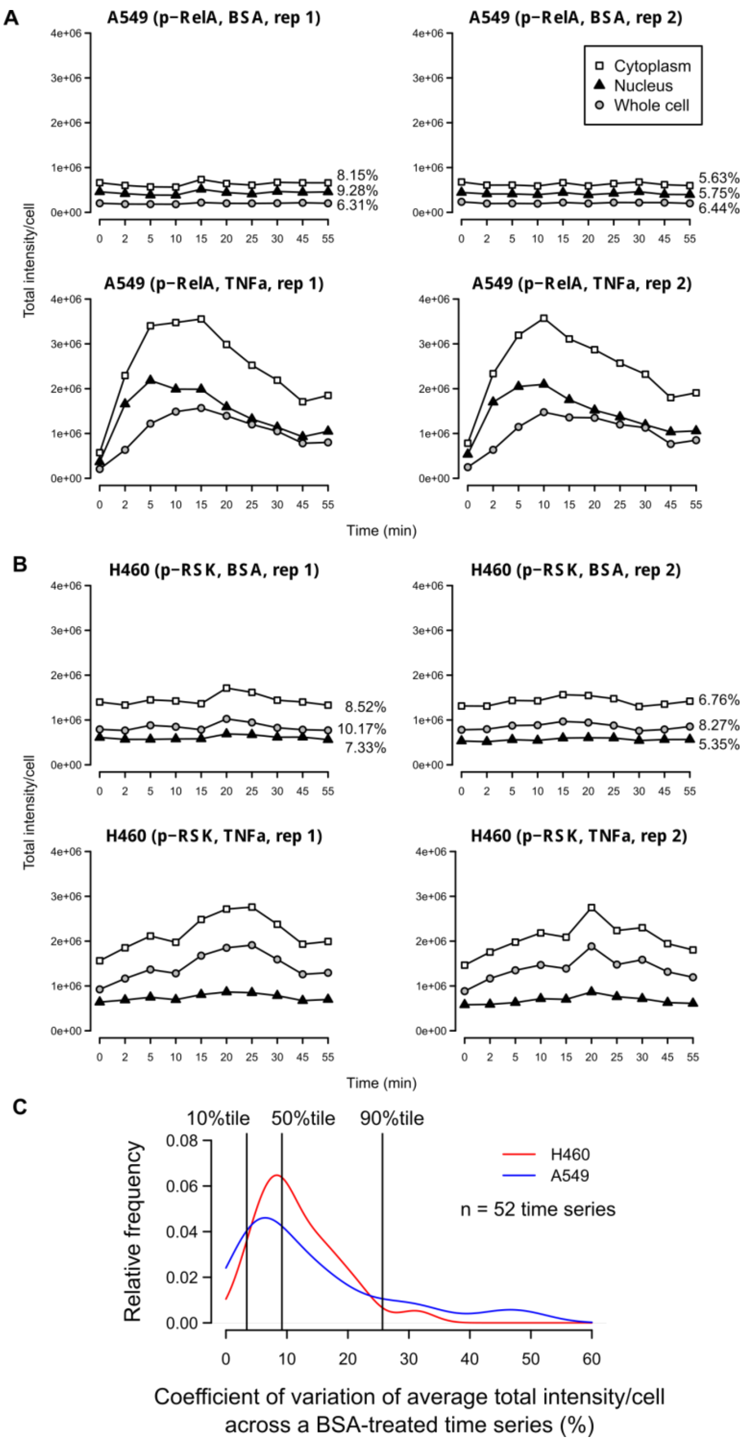

Figure S4. The raw fluorescence intensity levels of **A)** p-RelA^{S536} staining in A549 cells, or **B)** p-RSK^{Th573} staining in H460 cells treated with 0.1% BSA or 300 ng/mL of TNFα across different time points (or wells) in our assays. The coefficient of variation (CV) of each time series is shown next to the series, rep = replicate. **C)** Probability distribution functions of the CV values for all the time series of BSA-treated A549 (blue) or H460 (red) cells. (Vertical black lines = percentiles of the CV values across all the time series.)

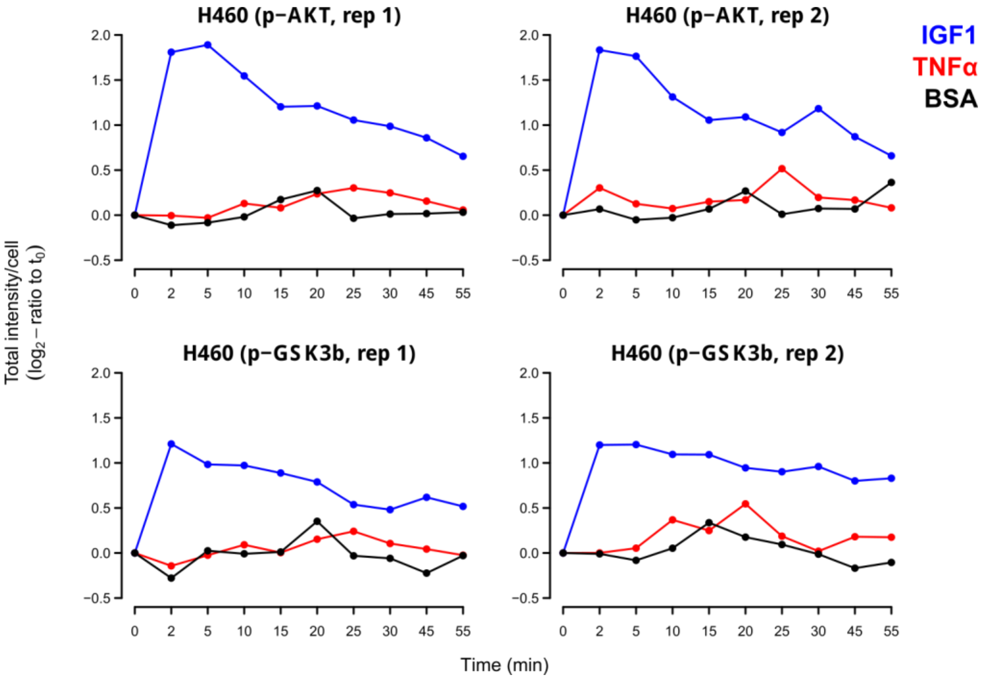

Figure S5. The normalized total intensity levels of p-AKT^{S473} and p-GSK3b^{S9} in H460 cells treated with 0.1% BSA (black), 300 ng/mL TNFα (red), or 300 ng/mL IGF1 (blue).

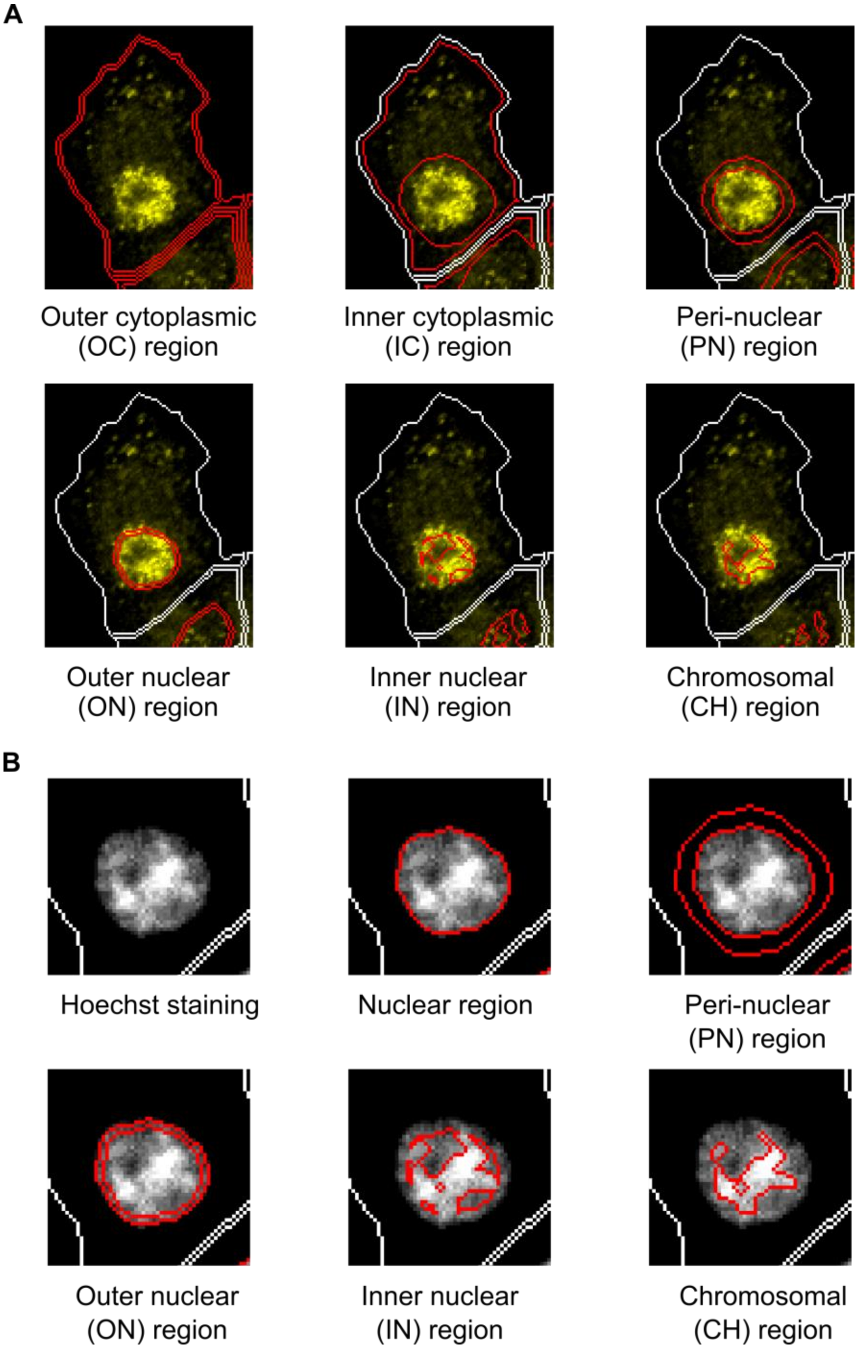

Figure S6. Examples of immunofluorescence images showing the different automatically detected subcellular regions (red boundaries) and whole-cell regions (white boundaries) overlaid with **A)** p-JNK$^{Th183/Ty185}$ (yellow) or **B)** Hoechst (white) staining. The whole-cell and nuclear regions were detected based on the COX-IV and Hoechst staining, respectively.

Supplementary Materials

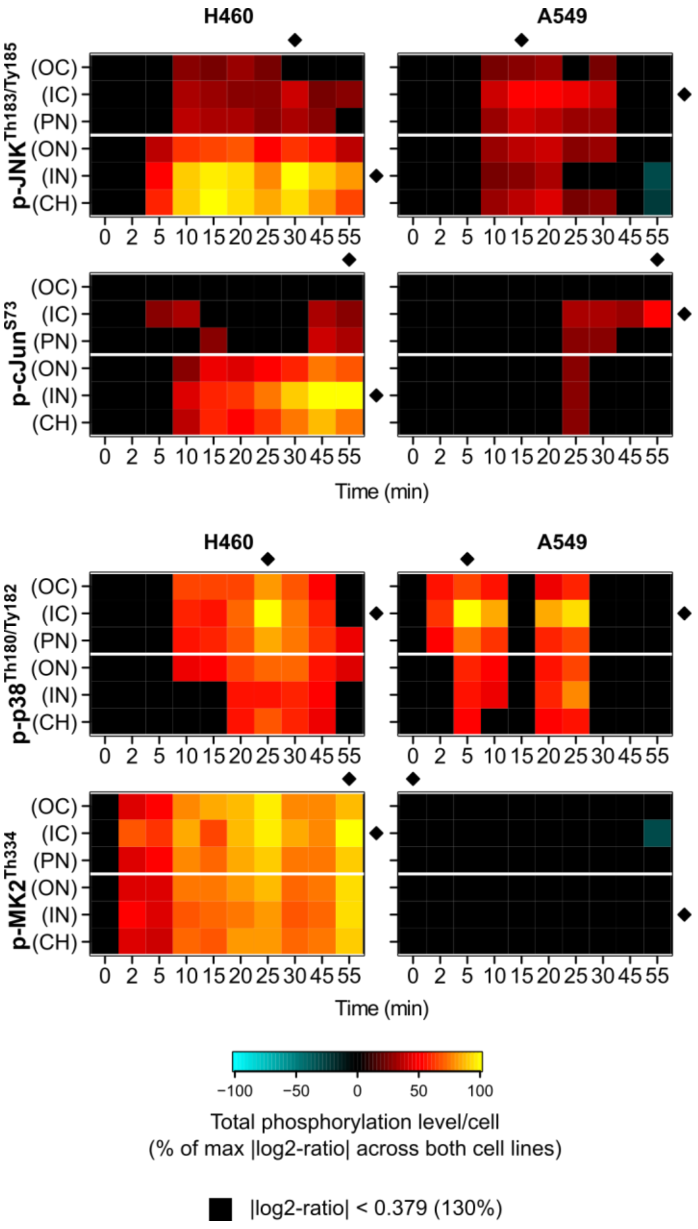

Figure S7. Heatmaps showing changes in the phosphorylation levels of two additional pairs of kinase-substrate signals at different subcellular regions in H460 and A549 cells treated with 300 ng/mL of TNFα. The values for all phosphorylation events are $\log_2$ ratios of their corresponding values at time 0 (without TNFα treatment). For visualization only, the $\log_2$ ratios for each signal are divided by their maximum absolute value across both cell lines in all regions (diamonds = subcellular regions or time points in which the maximum phosphorylation levels were detected).

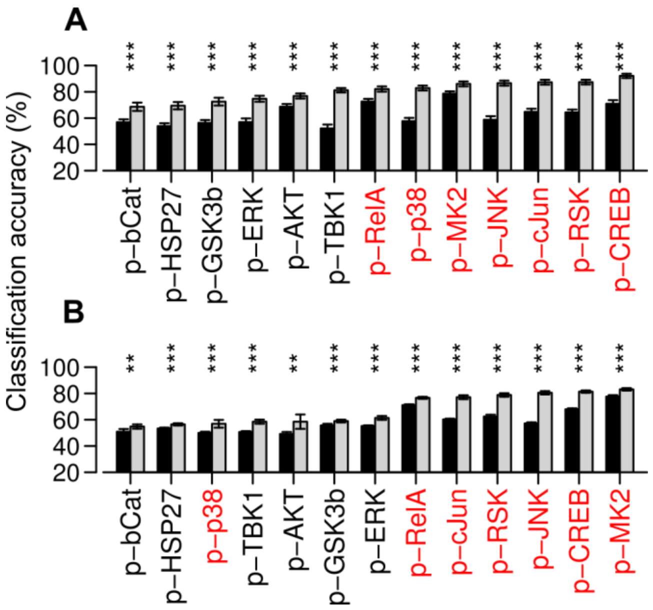

Figure S8. Mean balanced accuracy in classifying H460 and A549 cells using support vector machines based on the phosphorylation events of individual signals (red = signals selected for the second stage; ** = P<0.01, *** = P<0.001, two-sided t-test, n = 9; error bars = standard deviations). The values were estimated using **A)** a radial-basis-function kernel for the SVM and 10-fold cross validation, and **B)** a linear kernel for the SVM and 3-fold cross validation.

Supplementary Materials

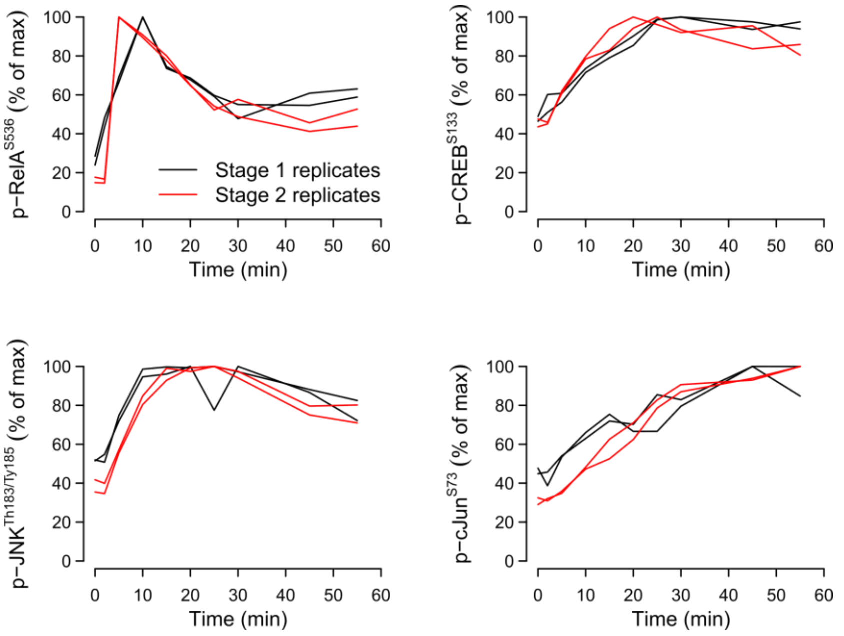

Figure S9. Examples of signaling responses curves obtained from H460 cells in the stages 1 and 2 experiments. Each experiment had two replicates, and each response curve was normalized by dividing all its values with the maximum value across all the time points.

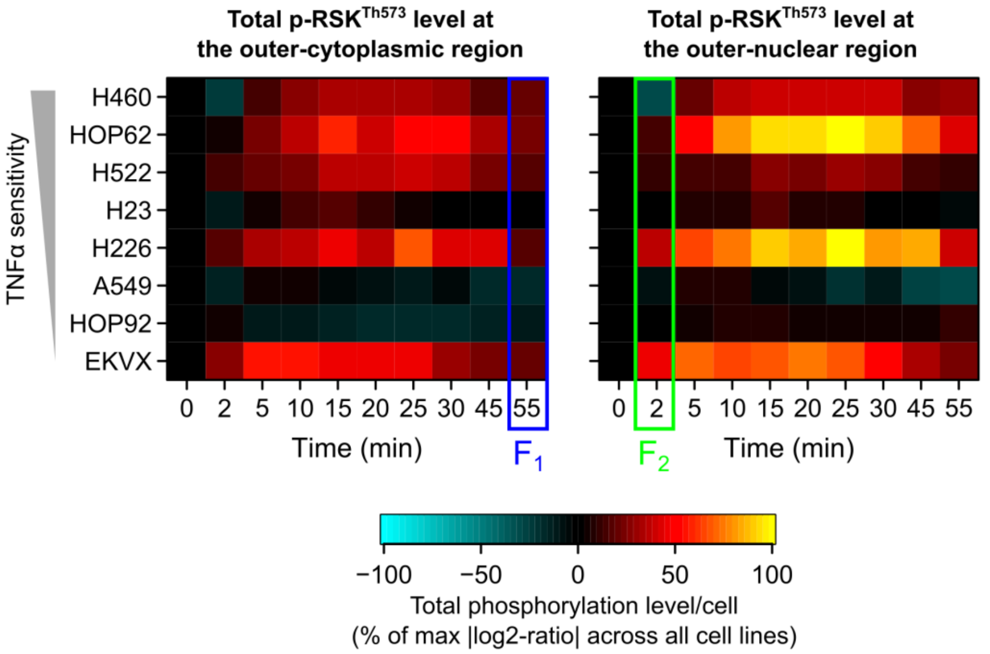

Figure S10. Heatmaps showing changes in the total phosphorylation levels of p-RSK^{Th573} at two different subcellular regions in all eight cell lines treated with 300 ng/mL of TNFα. The values for all phosphorylation events are $\log_2$ ratios of their corresponding values at time 0 (without TNFα treatment). For visualization only, the $\log_2$ ratios are divided by the maximum absolute value across all cell lines.

Supplementary Materials

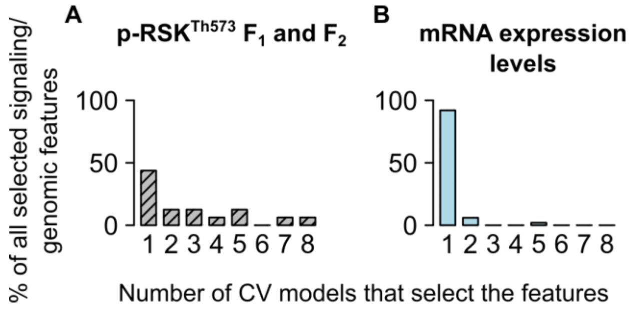

Figure S11. Distributions of the number of cross-validation (CV) models that commonly select a feature for models based on the **A)** p-RSK^{Th573} features, or **B)** mRNA expression levels of the NSCLC cell lines.

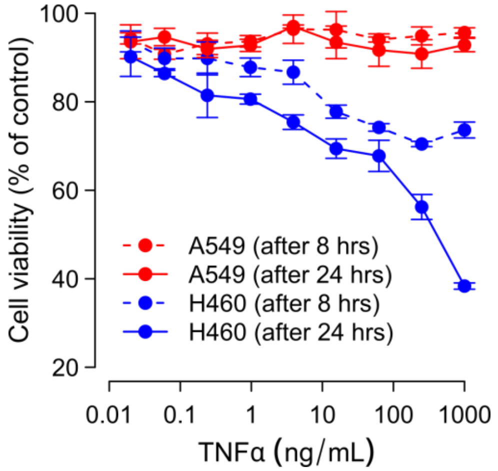

Figure S12. Dose response curves showing the percentages of viable A549 and H460 cells treated with different concentrations of TNFα for 8 or 24 hours (n=3, error bars=standard errors of the means). The values were obtained using the resazurin-based cell viability assay.

Supplementary Materials

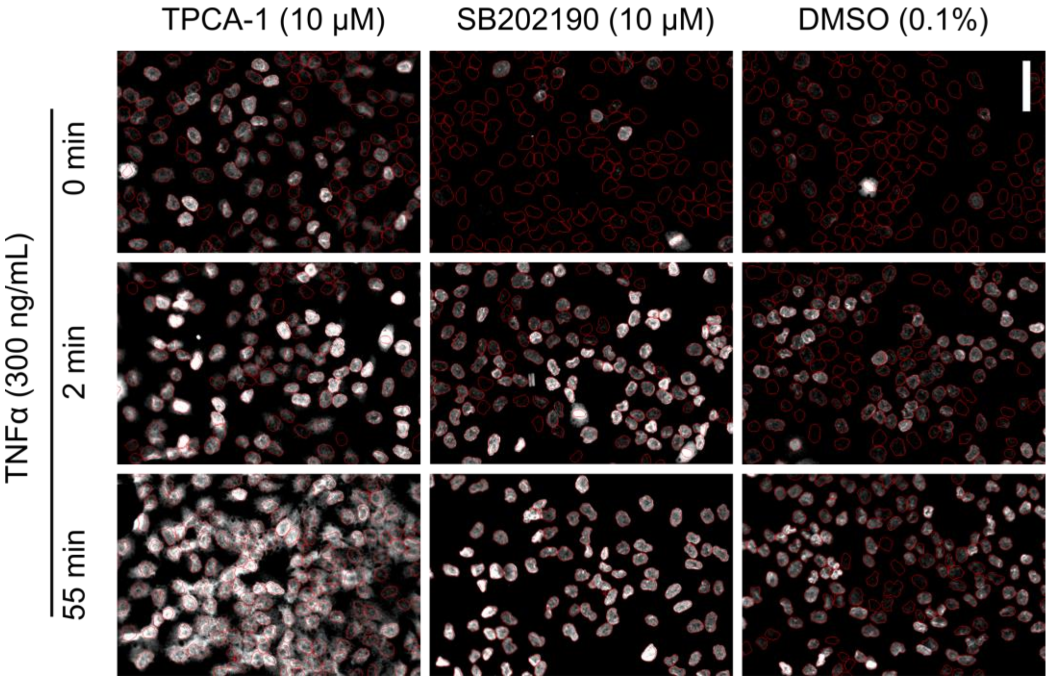

Figure S13. Immunofluorescence images showing the p-RSK^{Th573} staining of H460 cells pre-treated with either DMSO, TPCA-1, or SB202190, and then co-treated with TNFα (red lines = automatically determined nuclear boundaries, scale bar = 50 μm). All images have the same exposure times and display intensity ranges.

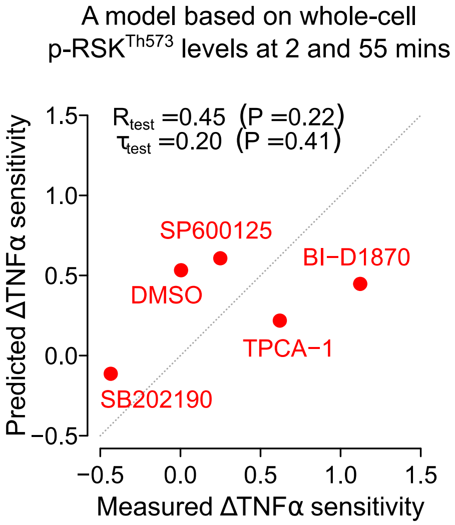

Figure S14. Scatter plot showing measured versus predicted ΔTNFα sensitivity indices of H460 cells treated with four different kinase inhibitors. The predictions were made using a regression model based on whole-cell p-RSK^{Th573} levels at 2 and 55 mins. (Dashed line = diagonal line, red = new test data that was not used to train the model, R_{test} = Pearson's correlation coefficient, τ_{test} = Kendalls' correlation coefficient, P-values shown were obtained from significance tests that the correlations are larger than zero).

Supplementary Materials

Table S1. List of the thirteen candidate intracellular signals in our study.

Protein (Symbol)	Phospho site	Relevance/ References
p65 subunit of nuclear factor-κB (p-RelA)	Serine 536	TNFα and other inflammatory cytokines activate RelA, a subunit of NF-κB [1]. NF-κB attenuates TNFα-induced cell death [2] and upregulates the expression of many pro-survival genes [3].
c-Jun N terminal kinase (p-JNK)	Threonine 183/ Tyrosine 185	JNK is phosphorylated and activated in response to stress and TNFα treatment [4]. TNFα-induced JNK activation is associated with the initiation of apoptosis [5,6].
Extracellular signal-regulated kinase (p-ERK)	Threonine 202/ Tyrosine 204	ERK is phosphorylated in response to numerous external stimuli, including TNFα [7]. TNFα may inhibit ERK activation triggered by receptor tyrosine kinase signals [8].
Tank binding kinase 1 (p-TBK1)	Serine 172	TBK1 interacts with TNF receptor-associated factor 2 (TRAF2) and TRAF binding protein (TANK) to promote TNFα-induced NF-κB activation [9].
p38 mitogen activated kinase (p-38)	Threonine 180/ Tyrosine 182	p38 is activated by dual phosphorylation at threonine 180 and tyrosine 182 in response to TNFα and other stress signals [10].
p90 ribosomal S6 kinase (p-RSK)	Threonine 573	RSK is phosphorylated at threonine 573 by ERK in response to mitogenic stimulation [11]. CREB is a RSK substrate [12].
cAMP response element- binding protein (p-CREB)	Serine 133	CREB is rapidly phosphorylated in response to TNFα treatment [13]. Serine 133 is a critical regulatory site that can be phosphorylated through p38 and ERK pathways [14].
Jun subunit of activating protein-1 transcription factor (p-cJun)	Serine 73	c-Jun can be phosphorylated at serine 63 and 73 by JNK [15]. TNFα-induced serine 73 phosphorylation increases c-Jun transcriptional activity and expression level [16].
MAP kinase activated protein kinase 2 (p-MK2)	Threonine 334	MK2 is activated by phosphorylations at threonine 334 and other residues after chemical stress [17].
Protein kinase B (p-AKT)	Serine 473	AKT can be activated by TNFα [18], and may be required for TNFα-induced activation of NF-κB [18,19].
Glycogen synthase kinase 3β (p-GSK3β)	Serine 9	GSK-3β is involved in both cell survival and death through the regulations of numerous factors, including NF-κB, βCat and CREB [20]. Inhibition of GSK-3β sensitizes hepatocytes to TNFα-induced apoptosis [21].
Beta catenin (p-βCat)	Serine 675	Phosphorylation of βCat at serine 675 by protein kinase A induces its nuclear accumulation and transcriptional activity [22]. βCat is negatively regulated by GSK-3β [23].
Heat shock protein 27 (p-HSP27)	Serine 82	TNFα and interleukin-1 treatments can stimulate HSP27 phosphorylation [24].

Supplementary Materials

Table S2. TNFα sensitivity values of the eight cell lines.

**Cell line**	EKVX	HOP92	A549	H226	H23	H522	HOP62	H460
**TNFα sensitivity**	0.0314	0.1480	0.1600	0.1720	0.3720	0.4510	0.5320	1.000

Table S3. Mutation status of the eight cell lines (from Ikediobi et. al., 2006).

**Cell lines**	**TP53**	**PTEN**	**EGFR**
**EKVX**	Mutated c.609_610GG>TT	Wild type	Wild type
**HOP92**	Mutated c.524G>T	Wild type	Wild type
**A549**	Wild type	Wild type	Wild type
**H226**	Inconclusive status	Wild type	Wild type
**H23**	Mutated c.738G>C	Wild type	Wild type
**H522**	Mutated c.572delC	Wild type	Wild type
**HOP62**	Mutated c.G673-2A>G	Wild type	Wild type
**H460**	Wild type	Wild type	Wild type

Supplementary Materials

References for Table S3

1. Baeuerle PA, Baltimore D. NF-kappa B: ten years after. Cell. 1996;87: 13–20.

2. Beg AA, Baltimore D. An essential role for NF-kappaB in preventing TNF-alpha-induced cell death. Science. 1996;274: 782–784.

3. Wang CY, Mayo MW, Korneluk RG, Goeddel DV, Baldwin AS. NF-kappaB antiapoptosis: induction of TRAF1 and TRAF2 and c-IAP1 and c-IAP2 to suppress caspase-8 activation. Science. 1998;281: 1680–1683.

4. Kyriakis JM, Banerjee P, Nikolakaki E, Dai T, Rubie EA, Ahmad MF, et al. The stress-activated protein kinase subfamily of c-Jun kinases. Nature. 1994;369: 156–160. doi:10.1038/369156a0

5. Guo Y-L. Correlation between Sustained c-Jun N-terminal Protein Kinase Activation and Apoptosis Induced by Tumor Necrosis Factor-alpha in Rat Mesangial Cells. J Biol Chem. 1998;273: 4027–4034. doi:10.1074/jbc.273.7.4027

6. Ventura J-J, Cogswell P, Flavell RA, Baldwin ASJ, Davis RJ. JNK potentiates TNF-stimulated necrosis by increasing the production of cytotoxic reactive oxygen species. Genes Dev. 2004;18: 2905–2915. doi:10.1101/gad.1223004

7. Geng Y, Valbracht J, Lotz M. Selective activation of the mitogen-activated protein kinase subgroups c-Jun NH2 terminal kinase and p38 by IL-1 and TNF in human articular chondrocytes. J Clin Invest. 1996;98: 2425–2430.

8. Muller G, Storz P, Bourteele S, Doppler H, Pfizenmaier K, Mischak H, et al. Regulation of Raf-1 kinase by TNF via its second messenger ceramide and cross-talk with mitogenic signalling. EMBO J. 1998;17: 732–742. doi:10.1093/emboj/17.3.732

9. Pomerantz JL, Baltimore, David. NF-kappa B activation by a signaling complex containing TRAF2, TANK and TBK1, a novel IKK-related kinase. EMBO J. 1999;18: 6694–6704. doi:10.1093/emboj/18.23.6694

10. Raingeaud J, Gupta S, Rogers JS, Dickens M, Han J, Ulevitch RJ, et al. Pro-inflammatory Cytokines and Environmental Stress Cause p38 Mitogen-activated Protein Kinase Activation by Dual Phosphorylation on Tyrosine and Threonine. J Biol Chem. 1995;270: 7420–7426. doi:10.1074/jbc.270.13.7420

11. Roux PP, Richards SA, Blenis J. Phosphorylation of p90 Ribosomal S6 Kinase (RSK) Regulates Extracellular Signal-Regulated Kinase Docking and RSK Activity. Mol Cell Biol. 2003;23: 4796–4804. doi:10.1128/MCB.23.14.4796-4804.2003

12. Frödin M, Gammeltoft S. Role and regulation of 90 kDa ribosomal S6 kinase (RSK) in signal transduction. Mol Cell Endocrinol. 1999;151: 65–77. doi:10.1016/S0303-7207(99)00061-1

13. Deak M, Clifton AD, Lucocq LM, Alessi DR. Mitogen- and stress-activated protein kinase-1 (MSK1) is directly activated by MAPK and SAPK2/p38, and may mediate activation of CREB. EMBO J. 1998;17: 4426–4441. doi:10.1093/emboj/17.15.4426

14. Xing J, Kornhauser JM, Xia Z, Thiele EA, Greenberg ME. Nerve growth factor activates extracellular signal-regulated kinase and p38 mitogen-activated protein kinase pathways to stimulate CREB serine 133 phosphorylation. Mol Cell Biol. 1998;18: 1946–1955.

15. Dérijard B, Hibi M, Wu I-H, Barrett T, Su B, Deng T, et al. JNK1: A protein kinase stimulated by UV light and Ha-Ras that binds and phosphorylates the c-Jun activation domain. Cell. 1994;76: 1025–1037. doi:10.1016/0092-8674(94)90380-8

16. Liu J, Minemoto Y, Lin A. c-Jun N-Terminal Protein Kinase 1 (JNK1), but Not JNK2, Is Essential for Tumor Necrosis Factor Alpha-Induced c-Jun Kinase Activation and Apoptosis. Mol Cell Biol. 2004;24: 10844–10856. doi:10.1128/MCB.24.24.10844-10856.2004

17. Ben-Levy R, Leighton IA, Doza YN, Attwood P, Morrice N, Marshall CJ, et al. Identification of novel phosphorylation sites required for activation of MAPKAP kinase-2. EMBO J. 1995;14: 5920–5930.

18. Nidai Ozes O, Mayo LD, Gustin JA, Pfeffer SR, Pfeffer LM, Donner DB. NF-κB activation by tumour necrosis factor requires the Akt serine–threonine kinase. Nature. 1999;401: 82–85. doi:10.1038/43466

19. Hatano E, Brenner DA. Akt protects mouse hepatocytes from TNF-α- and Fas-mediated apoptosis through NK-κB activation. Am J Physiol - Gastrointest Liver Physiol. 2001;281: G1357–G1368.

20. Hoeflich KP, Luo J, Rubie EA, Tsao MS, Jin O, Woodgett JR. Requirement for glycogen synthase kinase-3beta in cell survival and NF-kappaB activation. Nature. 2000;406: 86–90. doi:10.1038/35017574

21. Schwabe RF, Brenner DA. Role of glycogen synthase kinase-3 in TNF-α-induced NF-κB activation and apoptosis in hepatocytes. Am J Physiol - Gastrointest Liver Physiol. 2002;283: G204–G211. doi:10.1152/ajpgi.00016.2002

22. Taurin S, Sandbo N, Qin Y, Browning D, Dulin NO. Phosphorylation of β-Catenin by Cyclic AMP-dependent Protein Kinase. J Biol Chem. 2006;281: 9971–9976. doi:10.1074/jbc.M508778200

23. Yost C, Torres M, Miller JR, Huang E, Kimelman D, Moon RT. The axis-inducing activity, stability, and subcellular distribution of beta-catenin is regulated in Xenopus embryos by glycogen synthase kinase 3. Genes Dev. 1996;10: 1443–1454. doi:10.1101/gad.10.12.1443

24. Guesdon F, Freshney N, Waller RJ, Rawlinson L, Saklatvala J. Interleukin 1 and tumor necrosis factor stimulate two novel protein kinases that phosphorylate the heat shock protein hsp27 and beta-casein. J Biol Chem. 1993;268: 4236–4243.
